# Studies on the Selective Syntheses of Sodium Ditelluride and Dialkyl Ditellurides

**DOI:** 10.3390/molecules27248991

**Published:** 2022-12-16

**Authors:** Chorong Kim, Yoo Jin Lim, Ye Eun Kim, Hyunsung Cho, Sang Hyup Lee

**Affiliations:** College of Pharmacy and Innovative Drug Center, Duksung Women’s University, 33, Samyangro 144-gil, Dobong-gu, Seoul 01369, Republic of Korea

**Keywords:** tellurium, dialkyl ditelluride, dialkyl telluride, sodium ditelluride, sodium borohydride, tellurium reduction

## Abstract

Studies on the selective synthetic method for dialkyl ditellurides **1**, a representative class of organyl tellurium compounds, were presented. Considering the difficulty in conducting previous harsh reactions and in suppressing the formation of dialkyl tellurides **2** as side products, we optimized reaction conditions for selective syntheses of sodium ditelluride and the corresponding dialkyl ditellurides **1**. We reduced tellurium to sodium ditelluride by using NaBH_4_ and subsequently, treated the obtained sodium ditelluride with alkyl halides (RX) to give the target compounds **1**. Consequently, by applying various alkyl halides (RX) we achieved the selective syntheses of dialkyl ditellurides **1** (13 examples with 4 new compounds) in modest to good yields. We also suggested the mechanistic pathways to dialkyl ditellurides **1**.

## 1. Introduction

**Figure 1:**
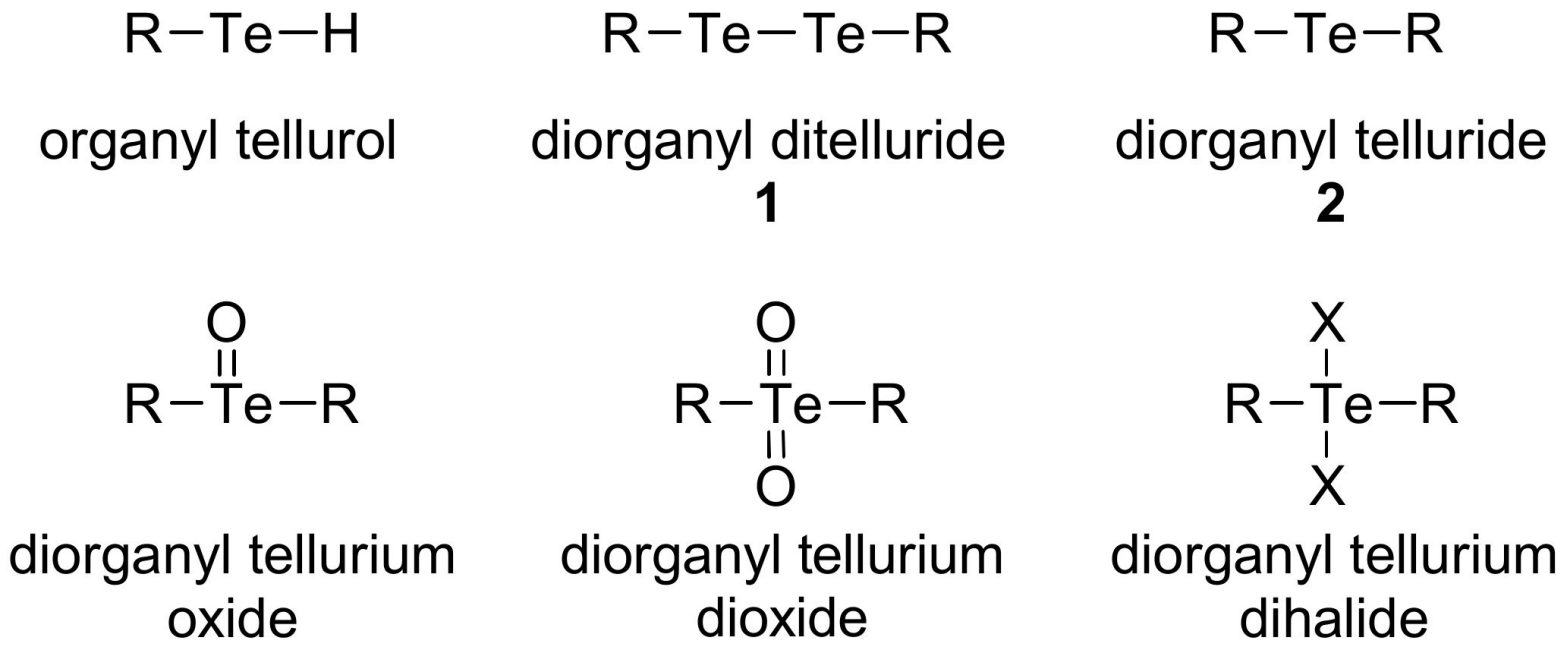
Types of organyl tellurium compounds.

Tellurium (Te), one of the chalcogens, is a chemically related element to sulfur (S) and selenium (Se). It exists in the crust of the earth at a concentration of 0.002 ppm and in similar oxidation states to selenium (−II, 0, IV, VI) [1,2,3]. Tellurium has often been compared to selenium and sulfur from the perspective of chemical properties and biological activities. For examples, the strengths of tellurium-carbon bonds (200 kJ/mol) are weaker than those of selenium-carbon bonds (234 kJ/mol), and the strengths of ditelluride bonds (149 kJ/mol) are also weaker than those of diselenide bonds (192 kJ/mol) [4]. It is also reported that heats of formation of Te_2−_ (ΔH_f_^o^_298.15_ = −8 kcal/mol) is smaller than those of Se_2−_ (ΔH_f_^o^_298.15_ = −11 kcal/mol) [5]. Tellurium has usually been employed in semiconductor industry, but its biochemical applications were relatively less highlighted [6]. In general, organyl chalcogen compounds have drawn attention due to their interesting chemical and medicinal properties. Among them, organyl selenium compounds have been considered attractive molecules that have important biological roles in the regulation of oxidative stresses [1,2,7]. As an extension, organyl tellurium compounds (Figure 1) have also been investigated due to their biochemical importance [8]. They are known to have various biological activities such as antimicrobial [9,10], antioxidant [11,12], and anti-tumor activities [8,13]. Interestingly, some organyl tellurium compounds have shown even higher biological activities than organyl selenium compounds [14]. However, there are not enough synthetic studies on organyl tellurium compounds compared to organyl selenium and sulfur compounds.

Among many organyl tellurium compounds, dialkyl ditellurides **1** are of our specific interest because we have already performed synthetic studies on diorganyl diselenides (R-Se-Se-R) that are similar to **1** [15]. In our preliminary studies, we found possibilities of selective syntheses of diorganyl ditellurides under mild reaction conditions, and thus focused on selectively synthesizing diorganyl ditellurides. So far, a few studies on synthetic methods of **1** using reducing agents have been reported. In general, elemental tellurium is treated with proper reducing agents, and then reacted with alkyl halides (RX) to give diorganyl ditellurides **1** [16,17,18,19,20]. Similar reactions were also performed by using potassium hydroxide (KOH) and alkyl halide under the presence of copper oxide (CuO) as a catalyst [21]. In other studies, strong nucleophilic reagents like alkyl lithium (RLi) [22,23] and Grignard reagent (RMgX) [24] also provided **1** by reacting with elemental tellurium. However, they usually provided dialkyl ditellurides **1** along with a substantial amount of dialkyl tellurides **2** as side products. Once dialkyl tellurides **2** (side products) are formed, it is really difficult to separate them because of their similar structure and properties. In the case of using nucleophilic reagents, it is hard to manipulate the harsh reaction conditions at extremely low temperature.

Based on our preliminary studies, we attempted to use sodium borohydride (NaBH_4_) or hydrazine hydrate (NH_2_NH_2_‧H_2_O) as the reducing agent. Herein, we report the selective and practical syntheses of sodium ditelluride (Na_2_Te_2_) and corresponding dialkyl ditellurides **1**. Furthermore, we investigated the mechanistic aspects for the generation of dialkyl ditellurides **1**.

## 2. Results and Discussion

### 2.1. Optimization

To perform the syntheses of dialkyl ditellurides **1**, we first investigated the reaction conditions for the selective formation of sodium ditelluride. So, we selected NaBH_4_ and NH_2_NH_2_‧H_2_O as reducing agents for elemental tellurium. However, since NH_2_NH_2_‧H_2_O gave inconclusive results in all of the reactions (data not shown), we finally decided to use NaBH_4_ as the appropriate reducing agent. A template alkyl halide was required to capture the sodium ditelluride in situ, and we chose 1-bromohexane (*n*-HexBr) according to our preliminary studies.

**Scheme 1:**
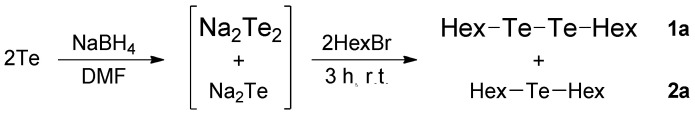
Formation of sodium ditelluride and di-*n*-hexyl ditelluride **1a**.

One-pot reactions were carried out using tellurium and NaBH_4_ in *N*,*N*-dimethylformamide (DMF) solvent. We optimized the reaction conditions by varying the amount of reducing agent, temperature, and time. The generated sodium ditelluride was reacted with *n*-HexBr, as shown in Scheme 1. To guarantee the reliability of the optimized conditions, we fixed the reaction condition for the subsequent alkylation step (1.2 eq of *n*-HexBr, 25 °C, and 3 h reaction time).

Although the crude product **1a** was obtained in reasonable yields in most of the reactions, the ratios of ditelluride **1a** to telluride **2a** were fluctuated depending on the reaction conditions (Table 1), which is consistent with our previous observations [15,25]. When NaBH_4_ was used in the range of 0.8–1.5 eq, the yields increased with the increasing amount of NaBH_4_, but the ratios of **1a** to **2a** decreased. The reaction temperature also influenced the results. However, when the reactions were conducted at 60–100 °C, the ratios of **1a** to **2a** tended to fluctuate at given amounts of NaBH_4_ and reaction times. Based on these results, we believed that the ditelluride dianion (Te_2_^2−^) could be further reduced to the telluride dianion (Te^2−^) that led to the formation of corresponding dihexyl telluride (**2a**). The reactions were also carried out in the range of time 0.5–2 h, and the ratios of **1a** to **2a** diminished over the elongated reaction time. This might imply that the formed ditelluride dianion could equilibrate with telluride dianion.

Considering the yields of the product and the selective formation of **1a** over **2a**, we chose the best condition as follows: Te (1.0 eq), NaBH_4_ (1.0 eq), and 1 h reaction time at 60 °C; HexBr (1.2 eq) and 3 h reaction time at 25 °C.

### 2.2. Syntheses of Dialkyl Ditellurides **1**

Using the optimized reaction conditions, we tried to synthesize dialkyl ditellurides **1** by using various aliphatic and aromatic halides. We used primary, secondary, and tertiary alkyl bromides. First, primary aliphatic halides gave good yields (76–84%, Table 2, entries 1–5 and 13). Then, secondary aliphatic halides gave modest to good yields (59–79%, Table 2, entries 6–12). Among them, *c*-hexyl bromide seemed to have a lower reactivity than the other ones, thus required longer reaction time (20 h) to give **1k** (Table 2, entry 11). Unfortunately, tertiary aliphatic halide such as *t*-butyl bromide gave no product, probably owing to steric hindrance by a bulky alkyl group. We also tried to use a sealed tube to avoid the vaporization of *t*-butyl bromide, which led to unsuccessful results. Aromatic bromides did not provide products, so we further tested aromatic iodides instead (e.g., phenyl, 4-nitrophenyl, 4-cyanophenyl, 4-methylphenyl, and 4-methoxyphenyl iodide). However, they simply gave mainly side products **2**. It seemed that the higher reaction temperature (110–153 °C) might lead to the facile formation of **2** due to the generated sodium telluride at high temperature. Notably, the reaction with benzyl bromide (BnBr) provided gradual formation of black solid during the work-up process. It seemed that the generated dibenzyl ditelluride might be decomposed to elemental tellurium, which derives some support from our previous observation [15]. Considering the high stability of analogous dibenzyl diselenide in our previous report [15], this observation seemed surprising. Given this fact, dibenzyl ditelluride could also be unstable while exposed to light and air, which requires further in-depth studies. Consequently, we succeeded in synthesizing dialkyl ditellurides (**1a**–**1m**) in moderate to good yields (59–84%) by using primary and secondary aliphatic bromides. Among them, four examples (**1g**–**1j**) are found new ditelluride compounds.

### 2.3. Studies on Reaction Pathways

**Scheme 2:**
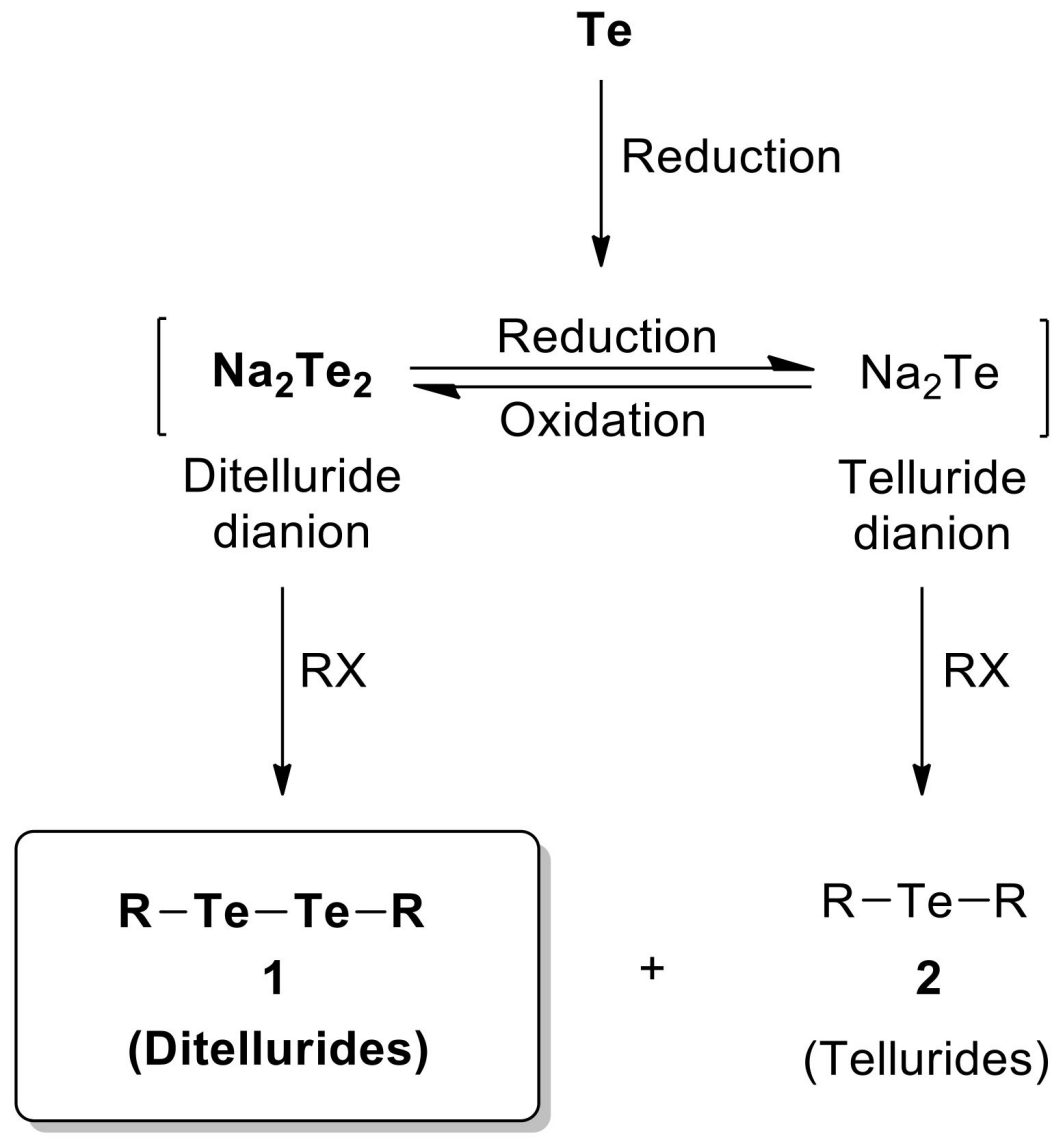
The proposed reaction pathways for products **1** and side products **2**.

Notably, we were interested in mechanistic studies on rection pathways. Herein, we present reaction pathways for the formation of dialkyl ditellurides **1** and dialkyl tellurides **2**, as shown in Scheme 2. At first, the elemental tellurium could be reduced to ditelluride dianion (Te_2_^2−^) by NaBH_4_, which could also be further reduced to telluride dianion (Te^2−^). The two dianions might equilibrate each other depending on reaction conditions such as the amount of NaBH_4_, reaction temperature, and reaction time. These species provided corresponding compounds, dialkyl ditellurides **1** and dialkyl tellurides **2**, respectively, by treating with alkyl halides (RX).

**Scheme 3:**
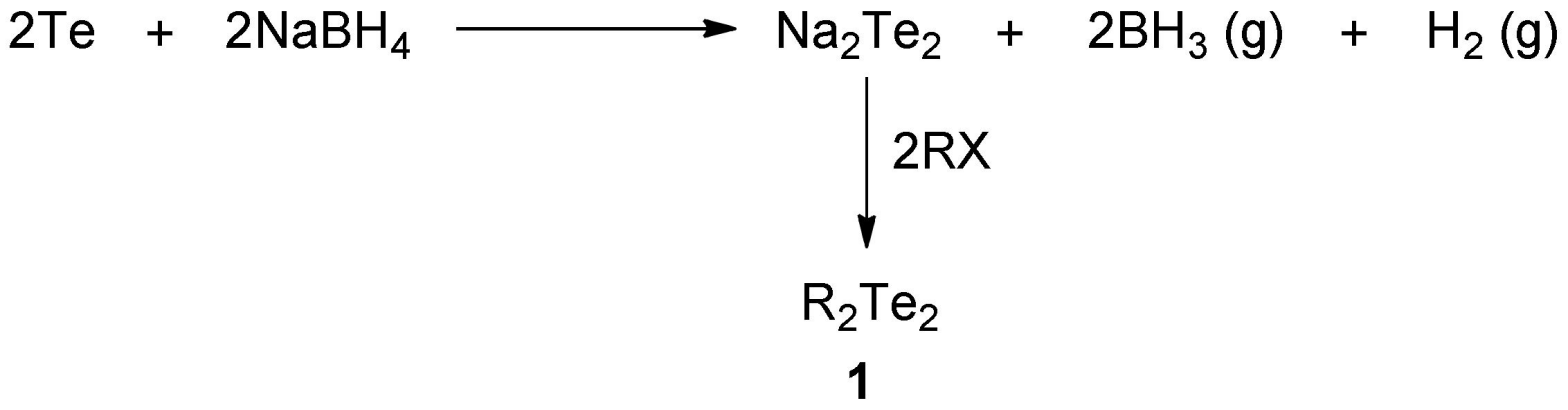
Reaction equation for sodium ditelluride and dialkyl ditellurides **1**.

Considering the theoretical amount of NaBH_4_ for the reduction of tellurium [26], we suggest chemical reactions for the formation of sodium ditelluride and corresponding dialkyl ditellurides **1,** as shown in Scheme 3. According to the chemical equation, the theoretical amount of NaBH_4_ is 1.0 eq compared to tellurium, and it affords 0.5 eq Na_2_Te_2_ that reacts with 1.0 eq alkyl halides to give dialkyl ditellurides **1**. In addition, borane (BH_3_) and hydrogen gas (H_2_) might be generated as byproducts in this process. Since selective syntheses of dialkyl ditellurides over dialkyl tellurides are always a significant issue, further in-depth studies are in progress.

## 3. Experimental

### 3.1. General Methods

The reagents were of commercial quality and used without further purification unless otherwise stated. Reactions were periodically monitored by thin-layer chromatography (TLC) (Merck & Co., Rahway, NJ, USA) carried on 0.25 mm Merck silica gel plates (60F-254) and visualized under UV light and *p*-anisaldehyde. Purifications were performed by column chromatography. Column chromatography was performed using Merck silica gels (230–400 mesh). Melting points (uncorrected) were determined in open capillary tubes using an Electrothermal IA9100 apparatus (Cole-Parmer, Vernon Hills, IL, USA). FT-IR spectra were recorded using a Thermo Fisher FT-IR spectrometer (Waltham, MA, USA). ^1^H (300 MHz) and ^13^C (75 MHz) NMR spectra were recorded on a Bruker DRX 300 spectrometer (Billerica, MA, USA) and chemical shifts (δ) are expressed relative to tetramethylsilane (TMS). Mass spectra were obtained in EI or ESI ionization modes. HPLC analyses were performed using the following Waters Associate Units: 1525 Binary HPLC pump, 2998 photodiode Array Detector, and C_18_ COSMOSIL column (4.6 × 250 mm^2^) (Waters, MA, USA). Reaction products were analyzed under gradient conditions: from 50% A (aqueous) and 50% B (MeCN) for 3 min (isocratic) to 10% A and 90% B in 20 min (gradient), then 10% A and 90% B for 30 min (isocratic). Finally, from 50% A and 50% B over 5 min (gradient), then keep 50% A and 50% B for 5 min (isocratic). The flow rate was 1 mL/min with eluent monitoring at 254 nm. HPLC solvents were filtered (aqueous solution with Millipore HVLP, 0.45 mm; MeCN with Millipore HV, 0.45 mm) and degassed before use.

### 3.2. General Procedure for the Syntheses of Dialkyl Ditellurides **1**

To a stirred mixture of NaBH_4_ (59 mg, 1.57 mmol, 1.0 eq) (TCI, Tokyo, Japan) in DMF (2.4 mL) (Daejung, Seoul, Korea) was added Te (200 mg, 1.57 mmol, 1.0 eq) (Alfa Aesar, Haverhill, MA, USA) under N_2_. The resulting mixture was stirred for 1 h at 60 °C during which it turned dark purple, indicating the formation of Na_2_Te_2_. The alkyl halide was added, and stirring was continued for 3–20 h at 25 °C until reaction completion (Table 2). The reaction mixture was then diluted with water (50 mL), extracted with *n*-hexane or ethyl acetate (2 × 50 mL), and washed with brine (50 mL). Combined organic layers were dried (anhydrous MgSO_4_) and concentrated in vacuo, forming a sticky residue that was purified by column chromatography (*n*-hexanes → 1:5 CH_2_Cl_2_/*n*-hexane) to give **1a**–**1m** as a red oil, unless otherwise noted. The charts for ^1^H- and ^13^C-NMR spectroscopies are available in Supplementary Materials.

#### 3.2.1. 1,2-Di-*n*-hexyl Ditelluride (**1a**)

Use of 1-bromohexane (263 μL, 1.9 mmol, 1.2 eq) (Sigma Aldrich, St. Louis, MO, USA) and 3 h reaction time at 25 °C in general procedure afforded the title compound **1a** (254 mg, 76%). Bp 153 °C; *R*_f_ 0.46 (*n*-hexane); HPLC t*_R_* 43.50 min; IR (ZnSe) 2956, 2923, 2853, 1465, 1154 cm^−1^; ^1^H NMR (300 MHz, CDCl_3_) *δ* 3.10 (t, *J* = 7.5 Hz, 4 H, TeCH_2_), 1.77–1.67 (m, 4 H, CH_2_), 1.41–1.25 (m, 12 H, CH_2_), 0.89 (t, *J* = 6.5 Hz, 6 H, CH_3_) [18]; ^13^C NMR (75 MHz, CDCl_3_) *δ* 33.8 (TeCH_2_), 31.4 (CH_2_), 31.3 (CH_2_), 22.8 (CH_2_), 14.3 (CH_2_), 4.8 (CH_3_); MS *m*/*z* 426 [M]^+^; HRMS (+EI) calcd for C_12_H_26_Te_2_ [M]^+^ 426.0129, found 426.0149 [18].

#### 3.2.2. 1,2-Di-*n*-butyl Ditelluride (**1b**)

Use of 1-bromobutane (203 μL, 1.9 mmol, 1.2 eq) (Acros, Waltham, MA, USA) and 3 h reaction time at 25 °C in general procedure afforded the title compound **1b** (235 mg, 81%). Bp 125 °C (lit [27]. 145–150 °C/5–6 mmHg); *R*_f_ 0.46 (*n*-hexane); HPLC t*_R_* 32.52 min; IR (ZnSe) 2956, 2923, 2870, 2856, 1463, 1158 cm^−1^; ^1^H NMR (300 MHz, CDCl_3_) *δ* 3.11 (t, *J* = 7.5 Hz, 4 H, TeCH_2_), 1.71 (quintet, *J* = 7.4 Hz, 4 H, CH_2_), 1.39 (sextet, *J* = 7.4 Hz, 8 H, CH_2_), 0.93 (t, *J* = 7.3 Hz, 6 H, CH_3_); ^13^C NMR (75 MHz, CDCl_3_) *δ* 35.7 (TeCH_2_), 24.6 (CH_2_), 13.4 (CH_2_), 4.3 (CH_3_); MS *m*/*z* 370 [M]^+^; HRMS (+EI) calcd for C_8_H_18_Te_2_ [M]^+^ 369.9502, found 369.9503. Spectral data were in accordance with literature information [27,28].

#### 3.2.3. 1,2-Di-*n*-pentyl Ditelluride (**1c**)

Use of 1-bromopentane (234 μL, 1.9 mmol, 1.2 eq) (Sigma Aldrich, St. Louis, MO, USA) and 3 h reaction time at 25 °C in general procedure afforded the title compound **1c** (250 mg, 80%). Bp 145 °C; *R*_f_ 0.50 (*n*-hexane); HPLC t*_R_* 44.83 min; IR (ZnSe) 2956, 2922, 2856, 1464, 1156 cm^−1^; ^1^H NMR (300 MHz, CDCl_3_) *δ* 3.10 (t, *J* = 7.6 Hz, 4 H, TeCH_2_), 1.78–1.68 (m, 4 H, CH_2_), 1.38–1.32 (m, 8 H, CH_2_), 0.90 (t, *J* = 7.0 Hz, 6 H, CH_3_) [29]; ^13^C NMR (75 MHz, CDCl_3_) *δ* 33.9 (TeCH_2_), 33.5 (CH_2_), 22.2 (CH_2_), 14.2 (CH_2_), 4.8 (CH_3_); MS *m*/*z* 398 [M]^+^; HRMS (+EI) calcd for C_10_H_22_Te_2_ [M]^+^ 397.9816, found 397.9825 [29].

#### 3.2.4. 1,2-Di-*n*-heptyl Ditelluride (**1d**)

Use of 1-bromoheptane (296 μL, 1.9 mmol, 1.2 eq) (Alfa Aesar, Haverhill, MA, USA) and 3 h reaction time at 25 °C in general procedure afforded the title compound **1d** (271 mg, 76%). Bp 164 °C; *R*_f_ 0.56 (*n*-hexane); HPLC t*_R_* 29.47 min; IR (ZnSe) 2956, 2922, 2852, 1466, 1151 cm^−1^; ^1^H NMR (300 MHz, CDCl_3_) *δ* 3.10 (t, *J* = 7.5 Hz, 4 H, TeCH_2_), 1.78–1.67 (m, 4 H, CH_2_), 1.33–1.28 (m, 16 H, CH_2_), 0.89 (t, *J* = 7.1 Hz, 6 H, CH_3_); ^13^C NMR (75 MHz, CDCl_3_) *δ* 33.9 (TeCH_2_), 32.0 (CH_2_), 31.7 (CH_2_), 28.8 (CH_2_), 22.9 (CH_2_), 14.3 (CH_2_), 4.8 (CH_3_); MS *m*/*z* 454 [M]^+^; HRMS (+EI) calcd for C_14_H_30_Te_2_ [M]^+^ 454.0442, found 454.0435. Spectral data were in accordance with literature information [21].

#### 3.2.5. 1,2-Di-*n*-octyl Ditelluride (**1e**)

Use of 1-bromooctane (325 μL, 1.9 mmol, 1.2 eq) (Sigma Aldrich, St. Louis, MO, USA) and 3 h reaction time at 25 °C in general procedure afforded the title compound **1e** (318 mg, 84%). Bp 175 °C; *R*_f_ 0.57 (*n*-hexane); HPLC t*_R_* 29.34 min; IR (ZnSe) 2956, 2923, 2852, 1465, 1151 cm^−1^; ^1^H NMR (300 MHz, CDCl_3_) *δ* 3.10 (t, *J* = 7.5 Hz, 4 H, TeCH_2_), 1.78–1.67 (m, 4 H, CH_2_), 1.38–1.21 (m, 24 H, CH_2_), 0.88 (t, *J* = 6.6 Hz, 6 H, CH_3_); ^13^C NMR (75 MHz, CDCl_3_) *δ* 33.9 (TeCH_2_), 32.1 (CH_2_), 31.7 (CH_2_), 29.4 (CH_2_), 29.1 (CH_2_), 22.9 (CH_2_), 14.3 (CH_2_), 4.8 (CH_3_); MS *m*/*z* 484 [M]^+^; HRMS (+EI) calcd for C_16_H_34_Te_2_ [M]^+^ 482.0745, found 482.0755. Spectral data were in accordance with literature information [27].

#### 3.2.6. 1,2-Di-*i*-propyl Ditelluride (**1f**)

Use of 2-bromopropane (177 μL, 1.9 mmol, 1.2 eq) (TCI, Tokyo, Japan) and 3 h reaction time at 25 °C in general procedure afforded the title compound **1f** (212 mg, 79%). Bp 56 °C; *R*_f_ 0.50 (*n*-hexane); HPLC t*_R_* 25.42 min; IR (ZnSe) 2962, 2942, 2912, 2855, 1454, 1190, 1142 cm^−1^; ^1^H NMR (300 MHz, CDCl_3_) *δ* 3.44 (quintet, *J* = 7.0 Hz, 2 H, TeCH), 1.62 (d, *J* = 7.0 Hz, 12 H, CH_3_) [17]; ^13^C NMR (75 MHz, CDCl_3_) *δ* 28.7 (TeCH), 10.2 (CH_3_); MS *m*/*z* 342 [M]^+^; HRMS (+EI) calcd for C_6_H_14_Te_2_ [M]^+^ 341.9189, found 341.9196 [17].

#### 3.2.7. 1,2-Bis(3-pentyl) Ditelluride (**1g**)

Use of 3-bromopentane (234 μL, 1.9 mmol, 1.2 eq) (TCI, Tokyo, Japan) and 3 h reaction time at 25 °C in general procedure afforded the title compound **1g** (237 mg, 76%). Bp 136 °C; *R*_f_ 0.49 (*n*-hexane); HPLC t*_R_* 40.02 min; IR (ZnSe) 2959, 2927, 2870, 2841, 1454, 1129 cm^−1^; ^1^H NMR (300 MHz, CDCl_3_) *δ* 3.13 (quintet, *J* = 6.6 Hz, 2 H, TeCH), 1.81–1.63 (m, 4 H, CH_2_), 0.98 (quintet, *J* = 7.37 Hz, 6 H, CH_3_); ^13^C NMR (75 MHz, CDCl_3_) *δ* 32.5 (TeCH), 31.5 (CH_2_), 14.2 (CH_3_); MS *m*/*z* 398 [M]^+^; HRMS (+EI) calcd for C_10_H_22_Te_2_ [M]^+^ 397.9816, found 397.9815.

#### 3.2.8. 1,2-Bis(4-heptyl) Ditelluride (**1h**)

Use of 4-bromoheptane (296 μL, 1.9 mmol, 1.2 eq) (Alfa Aesar, Haverhill, MA, USA) and 3 h reaction time at 25 °C in general procedure afforded the title compound **1h** (269 mg, 76%). Bp 133 °C; *R*_f_ 0.64 (*n*-hexane); HPLC t*_R_* 46.62 min; IR (ZnSe) 2956, 2927, 2871, 2833, 1463, 1377, 1130 cm^−1^; ^1^H NMR (300 MHz, CDCl_3_) *δ* 3.44 (quintet, *J* = 6.6 Hz, 2 H, TeCH), 1.74–1.33 (m, 16 H, CH_2_), 0.92 (t, *J* = 7.1 Hz, 12 H, CH_3_); ^13^C NMR (75 MHz, CDCl_3_) *δ* 41.3 (TeCH), 27.5 (CH_2_), 22.9 (CH_2_), 13.9 (CH_3_); MS *m*/*z* 454 [M]^+^; HRMS (+EI) calcd for C_14_H_30_Te_2_ [M]^+^ 454.0442, found 454.0440.

#### 3.2.9. 1,2-Di-*c*-butyl Ditelluride (**1i**)

Use of bromocyclobutane (177 μL, 1.9 mmol, 1.2 eq) (TCI, Tokyo, Japan) and 5 h reaction time at 25 °C in general procedure afforded the title compound **1i** (192 mg, 67%). Bp 103 °C; *R*_f_ 0.47 (*n*-hexane); HPLC t*_R_* 28.04 min; IR (ZnSe) 2969, 2931, 2857, 1246, 1173 cm^−1^; ^1^H NMR (300 MHz, CDCl_3_) *δ* 4.05–3.92 (m, 2 H, TeCH), 2.51–2.46 (m, 2 H, CH_2_), 2.39–2.03 (m, 8 H, CH_2_), 1.93–1.83 (m, 2 H, CH_2_); ^13^C NMR (75 MHz, CDCl_3_) *δ* 35.11 (TeCH), 23.07 (CH_2_), 9.24 (CH_2_); MS *m*/*z* 366 [M]^+^; HRMS (+EI) calcd for C_8_H_14_Te_2_ [M]^+^ 365.9189, found 365.9195.

#### 3.2.10. 1,2-Di-*c*-pentyl Ditelluride (**1j**)

Use of bromocyclopentane (202 μL, 1.9 mmol, 1.2 eq) (Sigma Aldrich, St. Louis, MO, USA) and 5 h reaction time at 25 °C in general procedure afforded the title compound **1j** (205 mg, 66%). Bp 130 °C; *R*_f_ 0.50 (*n*-hexane); HPLC t*_R_* 36.57 min; IR (ZnSe) 2951, 2863, 1446, 1191 cm^−1^; ^1^H NMR (300 MHz, CDCl_3_) *δ* 3.56 (quintet, *J* = 7.1 Hz, 2 H, TeCH), 2.08–1.97 (m, 4 H, CH_2_), 1.84–1.53 (m, 12 H, CH_2_); ^13^C NMR (75 MHz, CDCl_3_) *δ* 38.0 (TeCH), 25.4 (CH_2_), 18.0 (CH_2_); MS *m*/*z* 394 [M]^+^; HRMS (+EI) calcd for C_10_H_18_Te_2_ [M]^+^ 393.9502, found 393.9505.

#### 3.2.11. 1,2-Di-*c*-hexyl Ditelluride (**1k**)

Use of bromocyclohexane (232 μL, 1.9 mmol, 1.2 eq) (Sigma Aldrich, St. Louis, MO, USA) and 20 h reaction time at 25 °C in general procedure afforded the title compound **1k** (196 mg, 59%). Bp 156 °C; *R*_f_ 0.46 (*n*-hexane); HPLC t*_R_* 52.79 min; IR (ZnSe) 2923, 2847, 1445, 1167, 988 cm^−1^; ^1^H NMR (300 MHz, CDCl_3_) *δ* 3.39 (t, *J* = 10.6 Hz, 2 H, TeCH), 2.13–2.09 (m, 4 H, CH_2_), 1.66–1.56 (m, 10 H, CH_2_), 1.42–1.26 (m, 6 H, CH_2_); ^13^C NMR (75 MHz, CDCl_3_) *δ* 38.1 (TeCH), 28.5 (CH_2_), 25.9 (CH_2_), 22.5 (CH_2_) [30]; MS *m*/*z* 422 [M]^+^; HRMS (+EI) calcd for C_12_H_22_Te_2_ [M]^+^ 421.9816, found 421.9809 [30].

#### 3.2.12. 1,2-Di-*c*-heptyl Ditelluride (**1l**)

Use of bromocycloheptane (259 μL, 1.9 mmol, 1.2 eq) (Alfa Aesar, Haverhill, MA, USA) and 5 h reaction time at 25 °C in general procedure afforded the title compound **1l** (279 mg, 79%). Bp 147 °C; *R*_f_ 0.47 (*n*-hexane); HPLC t*_R_* 35.48 min; IR (ZnSe) 2921, 2849, 1455, 1196, 1177 cm^−1^; ^1^H NMR (300 MHz, CDCl_3_) *δ* 3.64–3.55 (m, 2 H, TeCH), 2.27–2.21 (m, 4 H, CH_2_), 1.84–1.73 (m, 4 H, CH_2_), 1.63–1.38 (m, 16 H, CH_2_); ^13^C NMR (75 MHz, CDCl_3_) *δ* 39.4 (TeCH), 28.4 (CH_2_), 28.1 (CH_2_), 25.4 (CH_2_) [30]; MS *m*/*z* 450 [M]^+^; HRMS (+EI) calcd for C_14_H_26_Te_2_ [M]^+^ 450.0129, found 450.0102 [30].

#### 3.2.13. 1,2-Bis(2-phenylethyl) Ditelluride (**1m**)

Use of (2-bromoethyl)benzene (257 μL, 1.9 mmol, 1.2 eq) (Sigma Aldrich, St. Louis, MO, USA) and 5 h reaction time at 25 °C in general procedure afforded the title compound **1m** (278 mg, 76%) as a dark yellow solid. M.p. 33 °C; *R*_f_ 0.14 (CH_2_Cl_2_: *n*-hexane = 1:5); HPLC t*_R_* 27.49 min; IR (ZnSe) 3023, 2923, 1493, 1452, 1145, 724, 696 cm^−1^; ^1^H NMR (300 MHz, CDCl_3_) *δ* 7.33–7.19 (m, 10 H, Ar), 3.33 (t, *J* = 7.9 Hz, 4 H, TeCH_2_), 3.06 (t, *J* = 8.0 Hz, 4 H, CH_2_) [31]; ^13^C NMR (75 MHz, CDCl_3_) *δ* 142.5 (Ar), 128.8 (Ar), 128.5 (Ar), 126.5 (Ar), 40.1 (TeCH_2_), 5.0 (CH_2_); MS *m*/*z* 467 [M]^+^; HRMS (+EI) calcd for C_16_H_18_Te_2_ [M]^+^ 465.9503, found 465.9491 [31].

## 4. Conclusions

We report an efficient method for the selective syntheses of sodium ditelluride and corresponding dialkyl ditellurides **1** over dialkyl tellurides **2**. We aimed to optimize reactions under mild conditions by avoiding the use of toxic reducing agents such as hydrazine. The optimized reaction conditions were as follows: (1) Te (1.0 eq), NaBH_4_ (1.0 eq) in DMF for 1 h at 60 °C; (2) alkyl bromides (1.2 eq) for 3–20 h at 25 °C. Using the optimized condition, we successfully achieved the selective syntheses of thirteen dialkyl ditellurides **1** without appreciable formation of dialkyl tellurides **2**, among which four compounds (**1g**–**1j**) are new. The primary aliphatic bromides gave **1a**–**1e**, and **1m** in good yields (76–84%) and the secondary bromides gave **1f**–**1l** in modest to good yields (59–79%). We also investigated the mechanistic studies on reaction pathways for the formation of sodium ditelluride and dialkyl ditellurides **1**.

## Figures and Tables

**Table 1:**
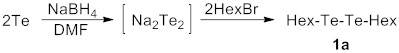
Optimization of reaction condition using NaBH_4_.

Entry	Na_2_Te_2_ Formation ^a^			Reaction with HexBr ^b^	Yields (%) (1a:2a) ^c^	Yields (%) (1a) ^c^
	NaBH_4_ (eq)	Temp (°C)	Time (h)	HexBr (eq)		
1	0.8	60	1	1.2	64 (11:1)	59
2		80	0.5		68 (24:1)	62
3	1.0	60	1		87 (14:1)	76
4		80	0.5		78 (13:1)	69
5	1.2	60	1		90 (2.3:1)	58
6		60	2		93 (1.8:1)	53
7		80	0.5		84 (2.3:1)	54
8		80	1		91 (4.0:1)	59
9		100	0.5		89 (2.0:1)	54
10		100	1		91 (4.0:1)	67
11	1.5	80	0.5		90 (0.76:1)	30

^a^ All reactions were run on 1.6 mmol scale of Te (1.0 eq, [c] = 670 mM) in DMF (2.4 mL); ^b^ 25 °C and 3 h reaction time; ^c^ Isolated yields.

**Table 2:**
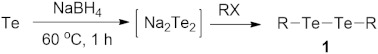
Syntheses of dialkyl ditellurides **1** ^a^.

Entry	Reaction with RX		Product	Yields (%) ^b^
	RX	Time (h)		
1	*n*-HexBr	3	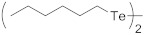 **1a**	76
2	*n*-BuBr	3	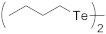 **1b**	81
3	*n*-PenBr	3	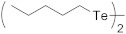 **1c**	80
4	*n*-HepBr	3	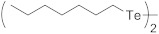 **1d**	76
5	*n*-OctBr	3	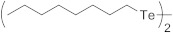 **1e**	84
6	*i*-PrBr	3	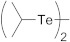 **1f**	79
7	3-Br-Pen	3	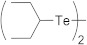 **1g**	76
8	4-Br-Hep	3	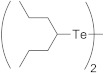 **1h**	76
9	*c*-BuBr	5	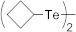 **1i**	67
10	*c*-PenBr	5	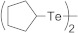 **1j**	66
11	*c*-HexBr	20	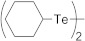 **1k**	59
12	*c*-HepBr	5	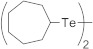 **1l**	79
13	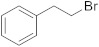	5	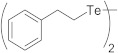 **1m**	76

^a^ All reactions were run on 1.6 mmol scale of Te (1.0 eq, [c] = 670 mM) in DMF (2.4 mL) and NaBH_4_ (1.0 eq) for 1 h at 60 °C for formation of Na_2_Te_2_; RBr (1.0 eq) at 25 °C for formation of **1**; ^b^ Isolated yields.

## Data Availability

The data presented in this study are available in insert article or Supplementary Materials.

## Supplementary Materials

The following supporting information can be downloaded at: https://www.mdpi.com/article/10.3390/molecules27248991/s1. Figures S1–S13 are the charts for ^1^H- and ^13^C-NMR spectroscopies.

## Sample Availability

Samples of the compounds **1a**–**1m** are available from the authors.
