# Supplementary material for: Studies on the Selective Syntheses of Sodium Ditelluride and Dialkyl Ditellurides
Source: Molecules. 2022 Dec 16;27(24):8991. doi: 10.3390/molecules27248991 (PMC9782605; doi:10.3390/molecules27248991)
Supplement: Supplementary file 1 [file molecules-27-08991-s001.zip › molecules-2099438-supplementary.pdf]

Supplementary data

for

Studies on the Selective Synthesis of Disodium Ditelluride and Diorganyl Ditellurides

Chorong Kim, Yoo Jin Lim, Ye Eun Kim, Hyunsung Cho, and Sang Hyup Lee *

College of Pharmacy and Innovative Drug Center, Duksung Women's University, Seoul 01369, Republic of Korea,

*sanghyup@duksung.ac.kr

Figure S1. ^{1}H and ^{13}C NMR spectrum of compound **1a** S2
Figure S2. ^{1}H and ^{13}C NMR spectrum of compound **1b** S3
Figure S3. ^{1}H and ^{13}C NMR spectrum of compound **1c** S4
Figure S4. ^{1}H and ^{13}C NMR spectrum of compound **1d** S5
Figure S5. ^{1}H and ^{13}C NMR spectrum of compound **1e** S6
Figure S6. ^{1}H and ^{13}C NMR spectrum of compound **1f** S7
Figure S7. ^{1}H and ^{13}C NMR spectrum of compound **1g** S8
Figure S8. ^{1}H and ^{13}C NMR spectrum of compound **1h** S9
Figure S9. ^{1}H and ^{13}C NMR spectrum of compound **1i** S10
Figure S10. ^{1}H and ^{13}C NMR spectrum of compound **1j** S11
Figure S11. ^{1}H and ^{13}C NMR spectrum of compound **1k** S12
Figure S12. ^{1}H and ^{13}C NMR spectrum of compound **1l** S13
Figure S13. ^{1}H and ^{13}C NMR spectrum of compound **1m** S14

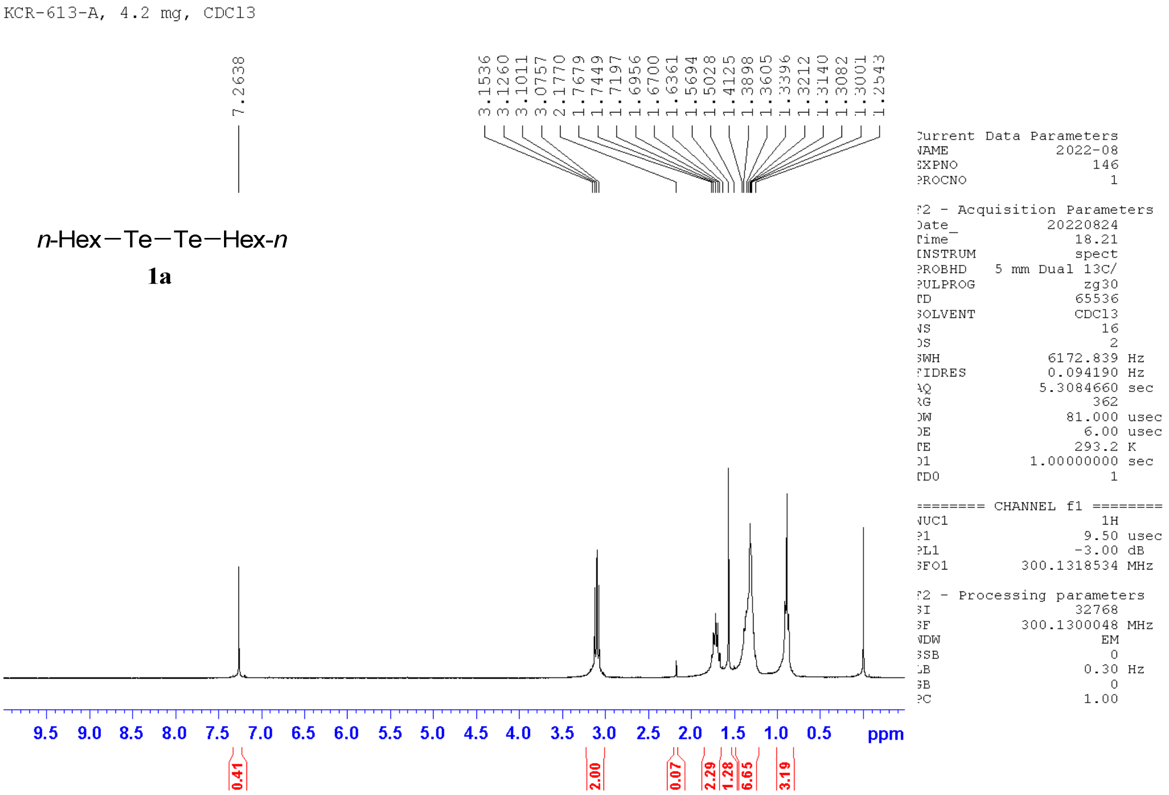

Figure S1a. ^{1}H NMR spectrum (300 MHz, $CDCl_3$) of compound **1a.**

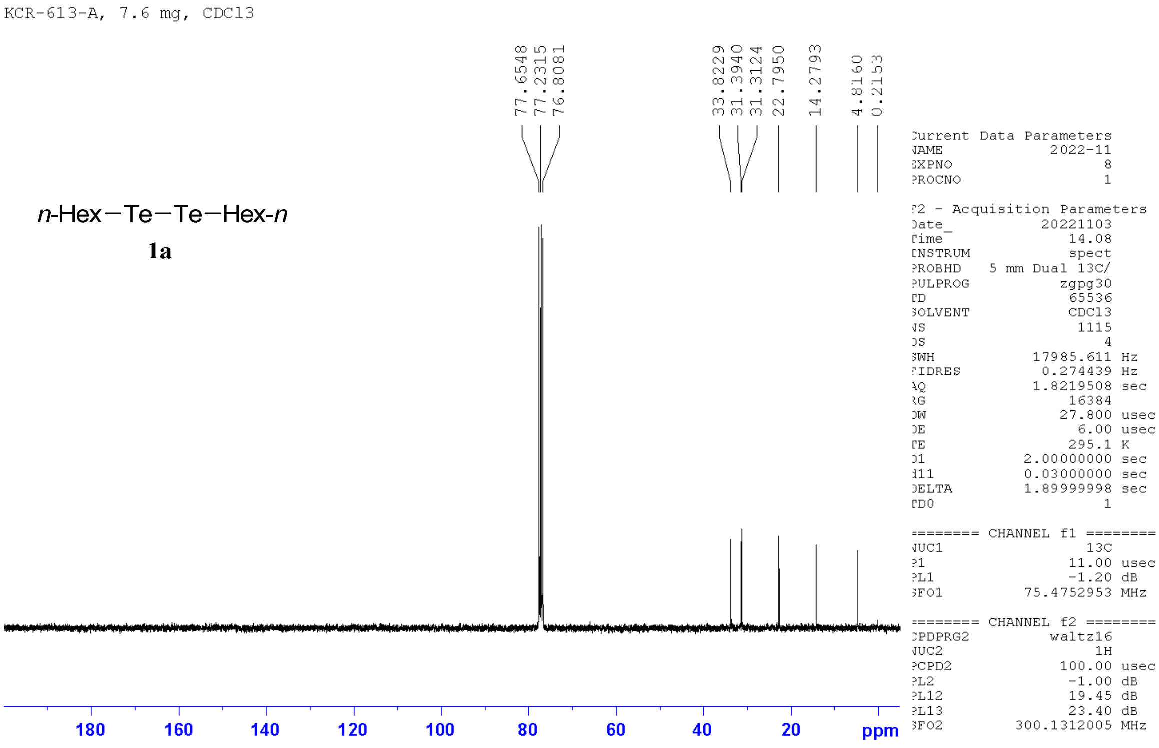

Figure S1b. ^{13}C NMR spectrum (75 MHz, $CDCl_3$) of compound **1a.**

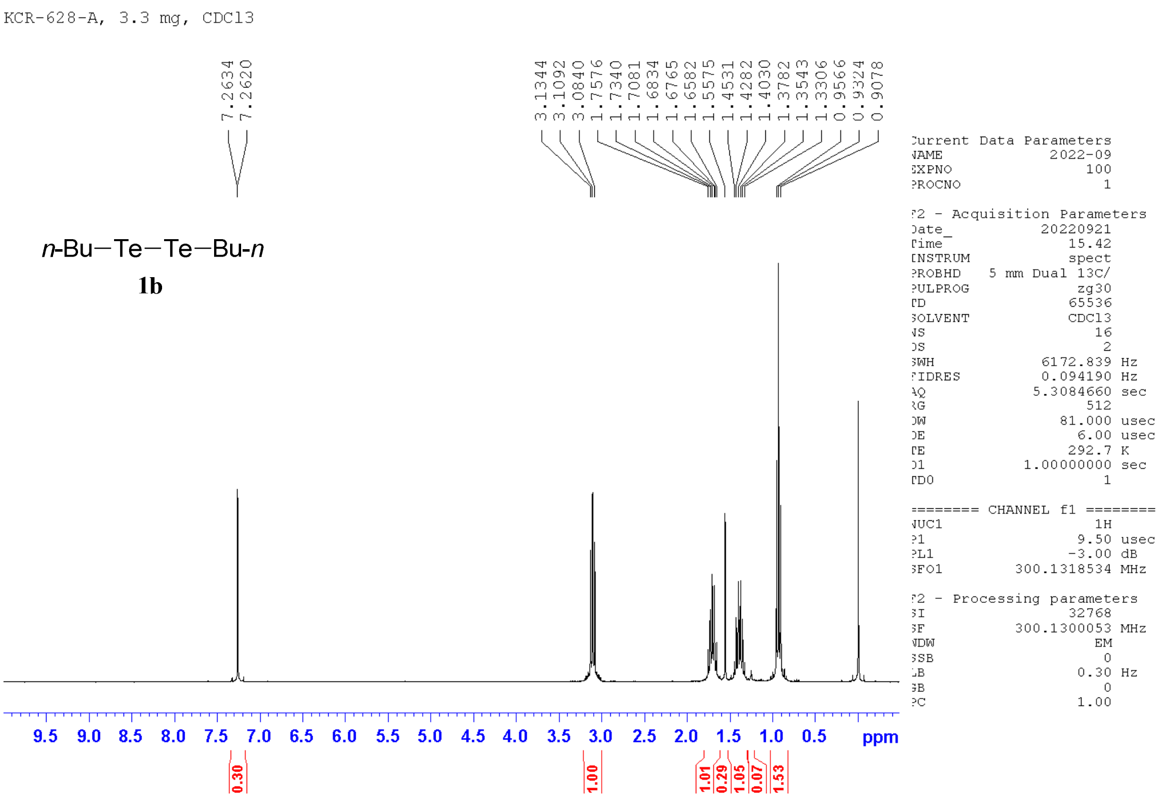

Figure S2a. ^{1}H NMR spectrum (300 MHz, $CDCl_3$) of compound **1b.**

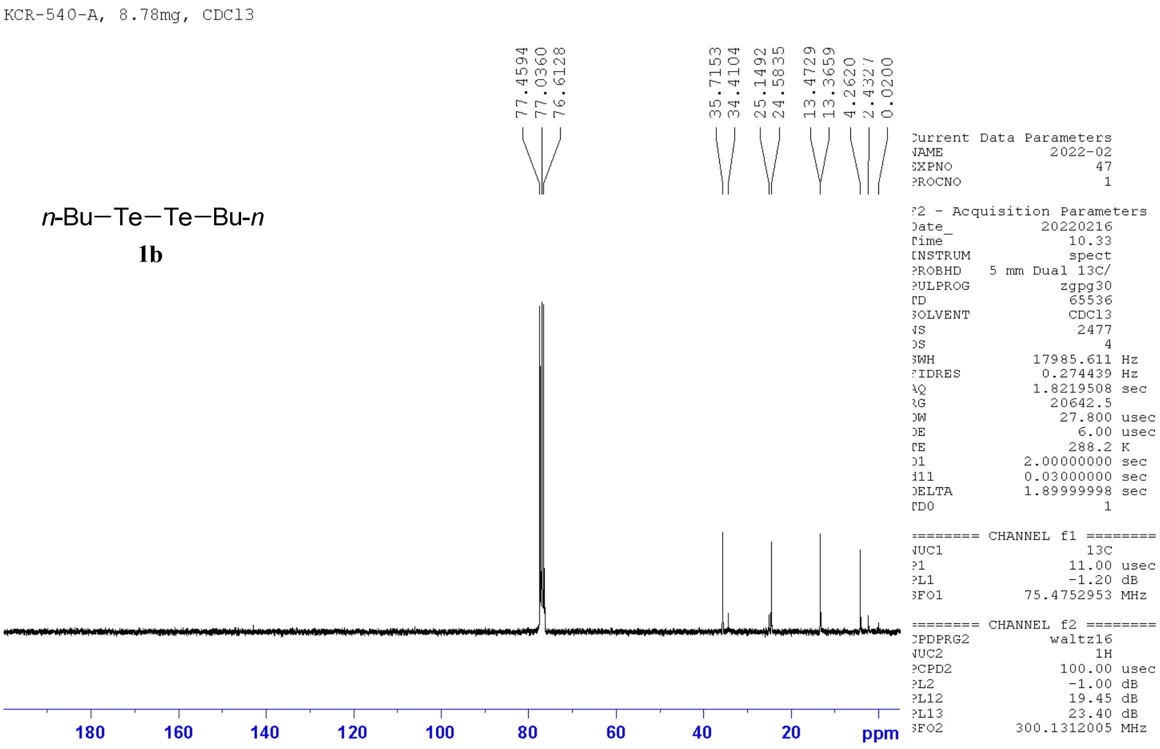

Figure S2b. ^{13}C NMR spectrum (75 MHz, $CDCl_3$) of compound **1b.**

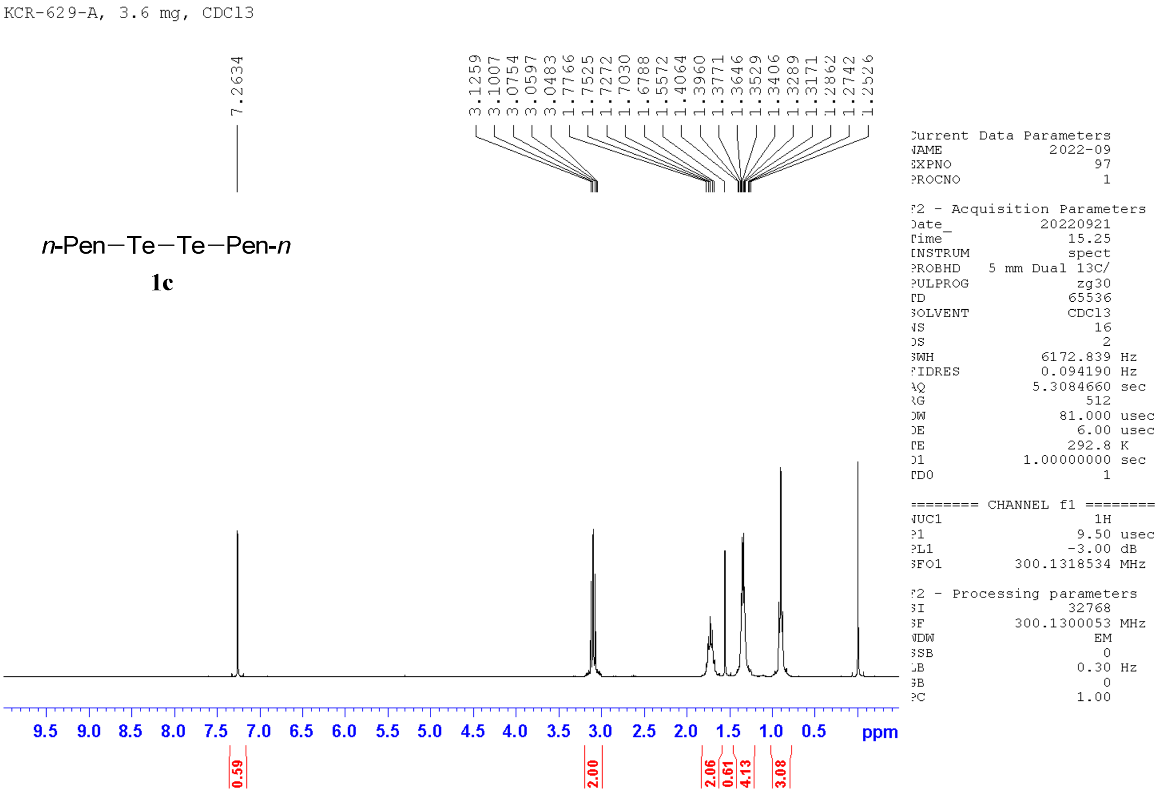

Figure S3a. ^{1}H NMR spectrum (300 MHz, $CDCl_3$) of compound **1c.**

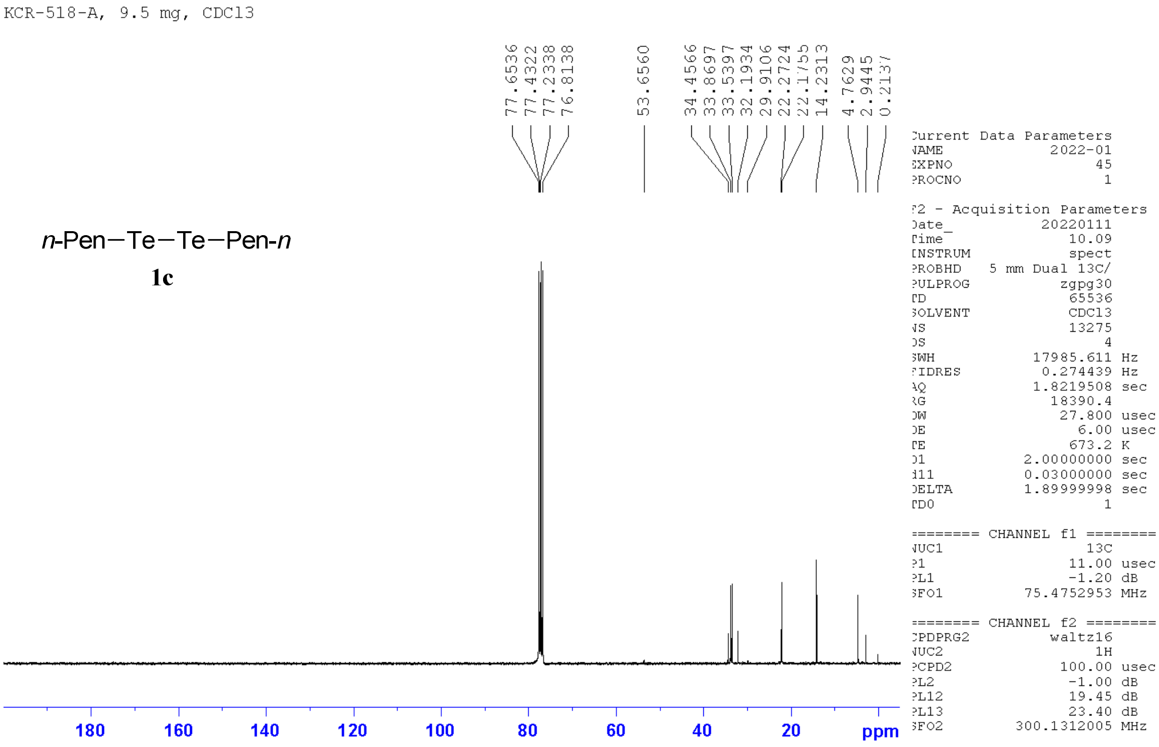

Figure S3b. ^{13}C NMR spectrum (75 MHz, $CDCl_3$) of compound **1c.**

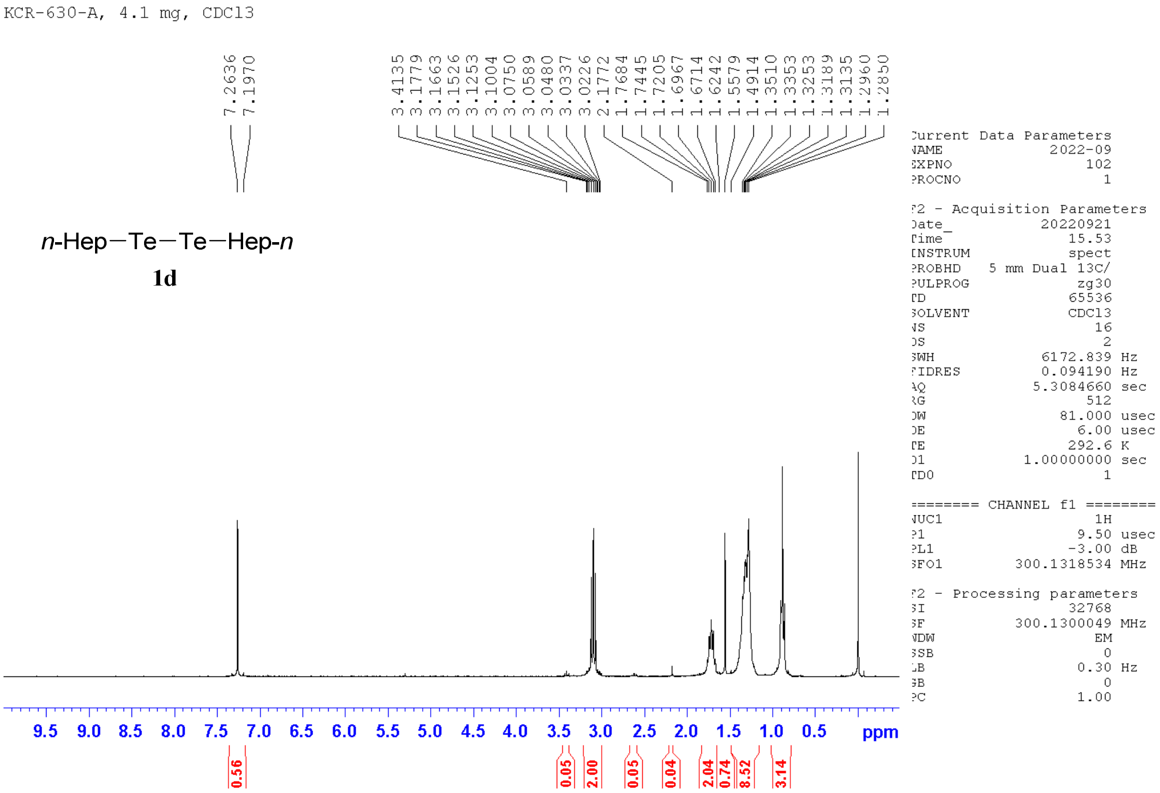

Figure S4a. ^{1}H NMR spectrum (300 MHz, $CDCl_3$) of compound **1d.**

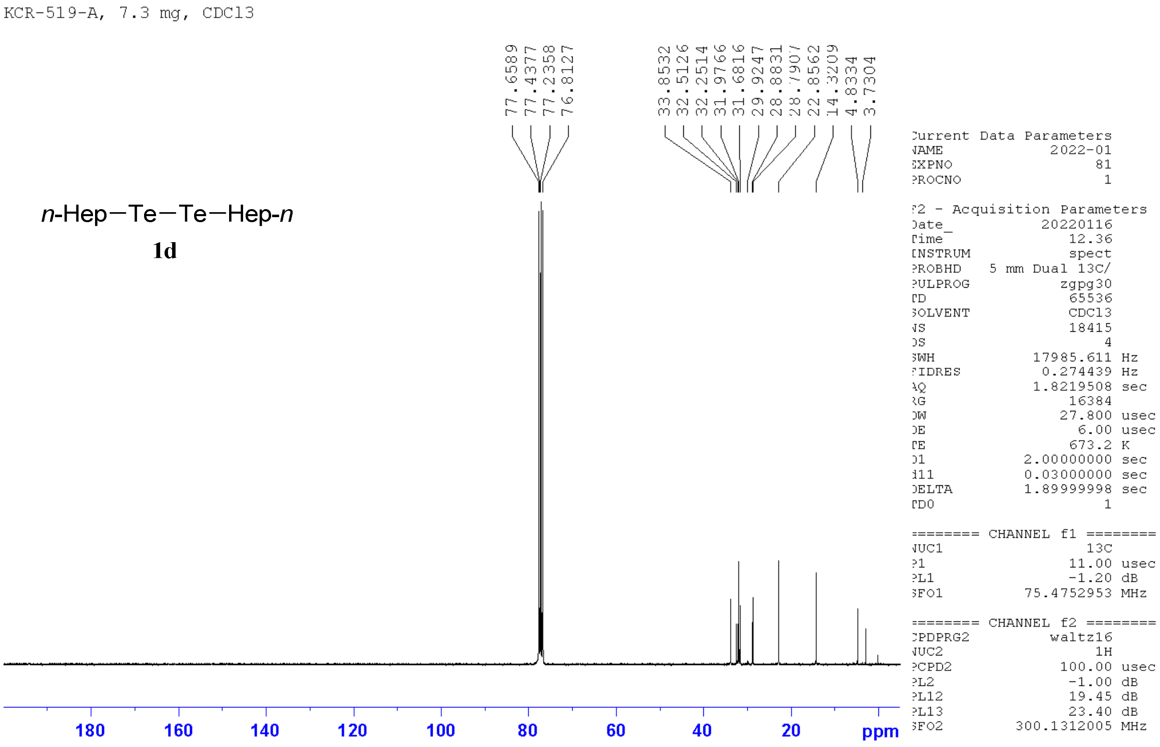

Figure S4b. ^{13}C NMR spectrum (75 MHz, $CDCl_3$) of compound **1d.**

KCR-523-A, 3.6 mg, CDCl3

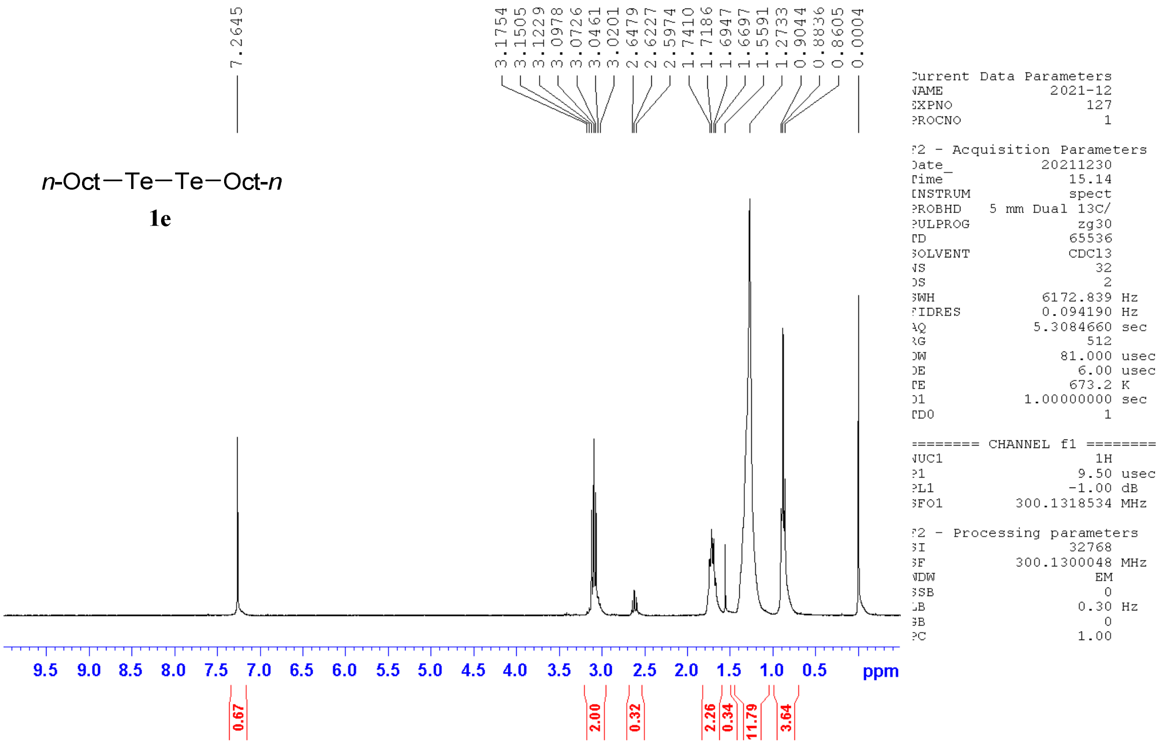

Figure S5a. ^{1}H NMR spectrum (300 MHz, $CDCl_3$) of compound **1e.**

KCR-523-A, 7.0 mg, CDCl3

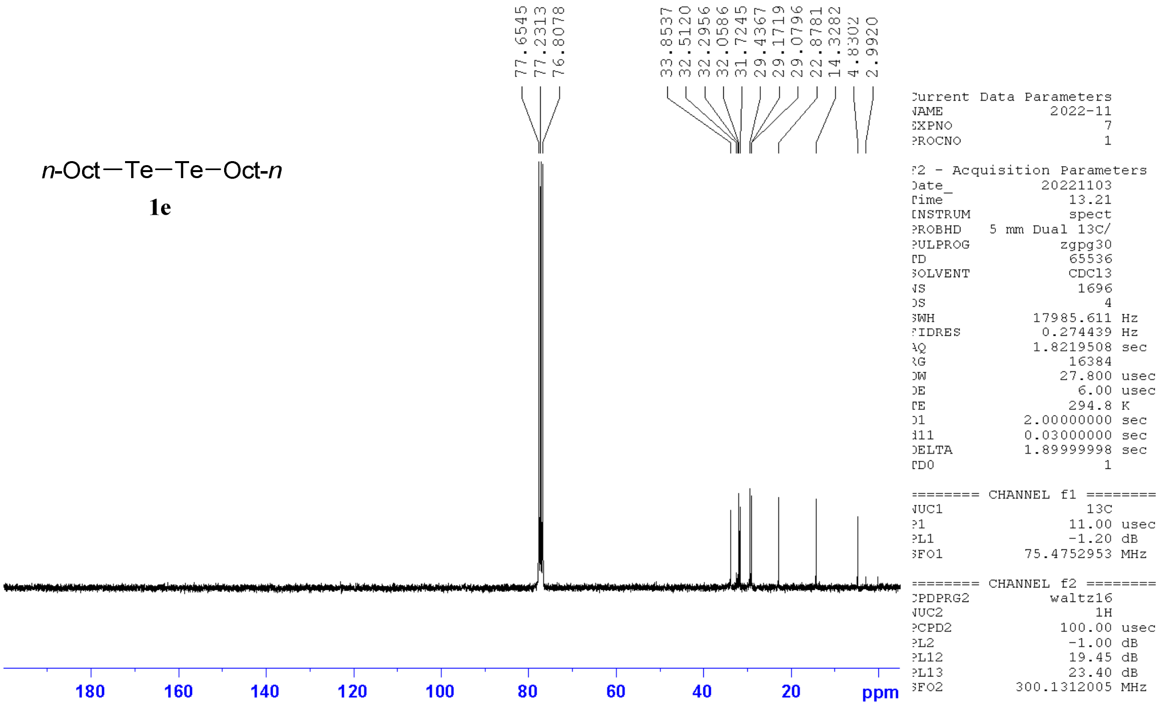

Figure S5b. ^{13}C NMR spectrum (75 MHz, $CDCl_3$) of compound **1e.**

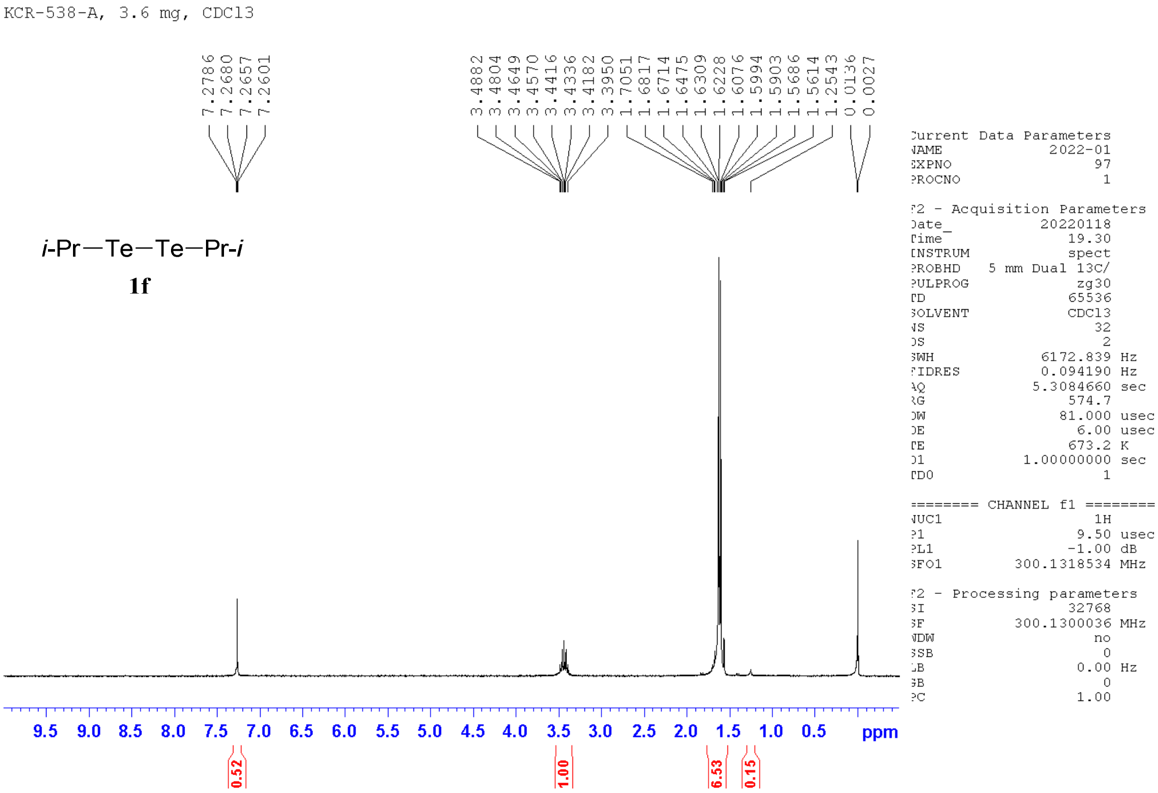

Figure S6a. ^{1}H NMR spectrum (300 MHz, $CDCl_3$) of compound **1f.**

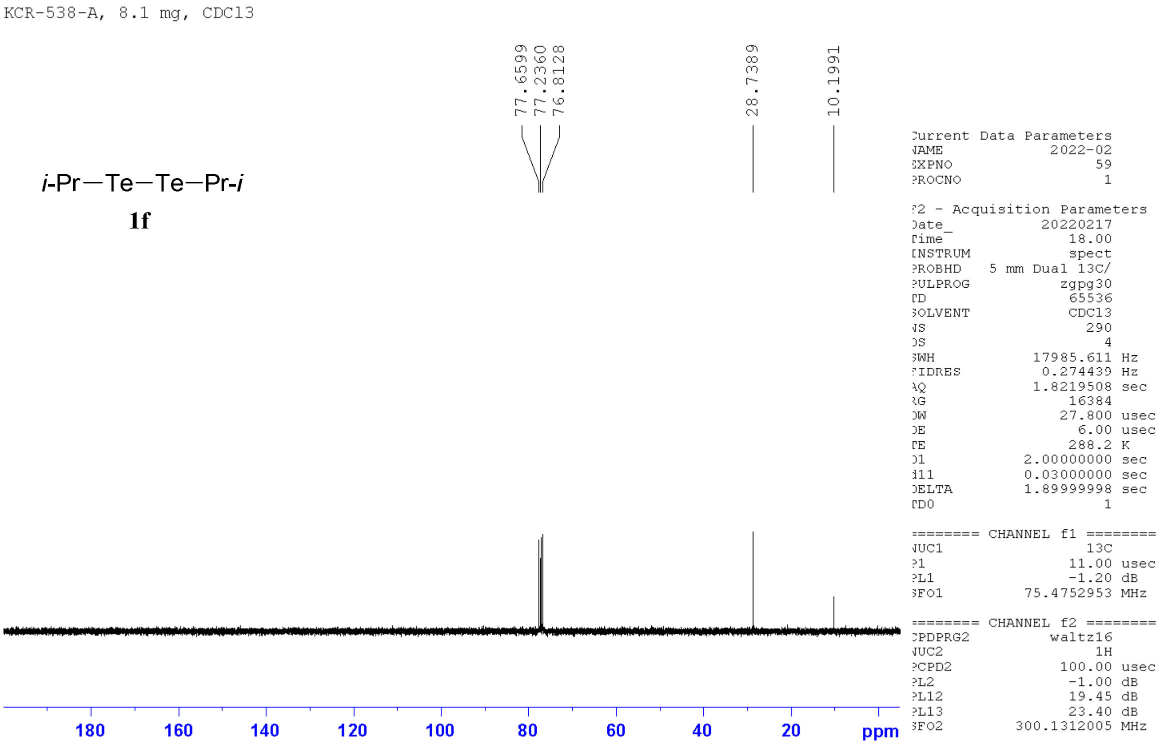

Figure S6b. ^{13}C NMR spectrum (75 MHz, $CDCl_3$) of compound **1f.**

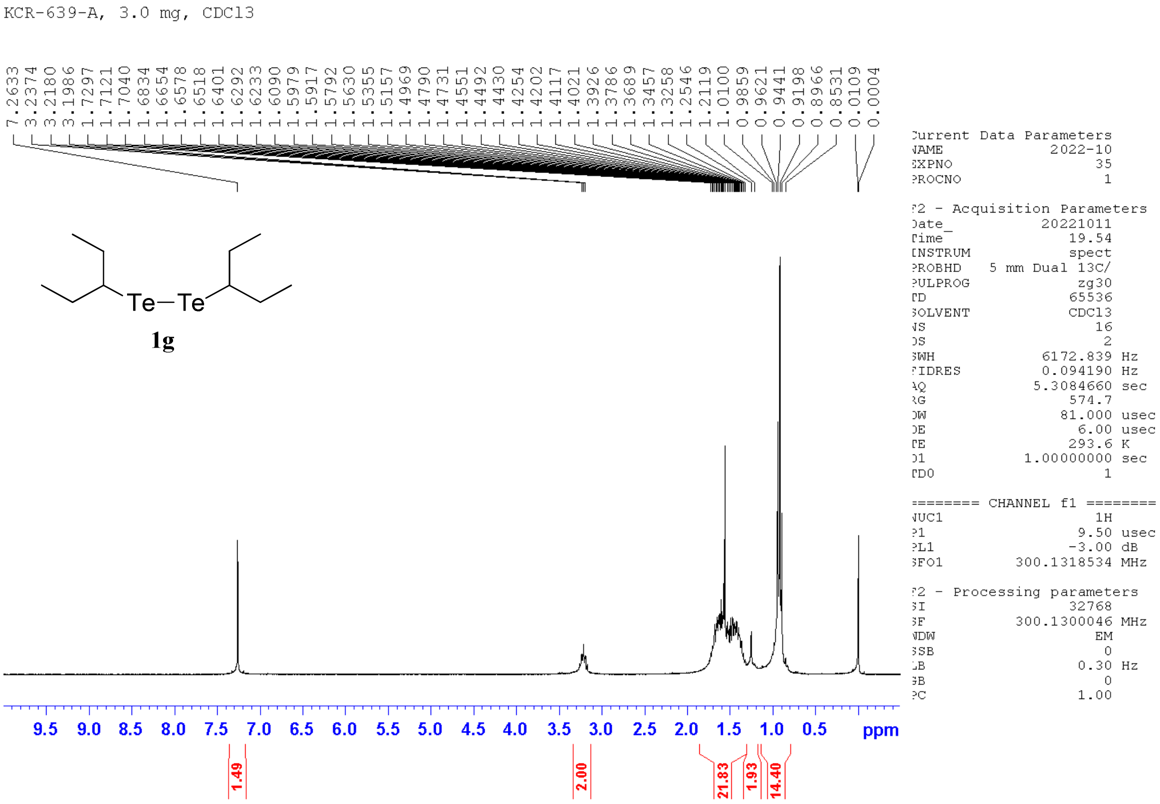

Figure S7a. ^{1}H NMR spectrum (300 MHz, $CDCl_3$) of compound **1g.**

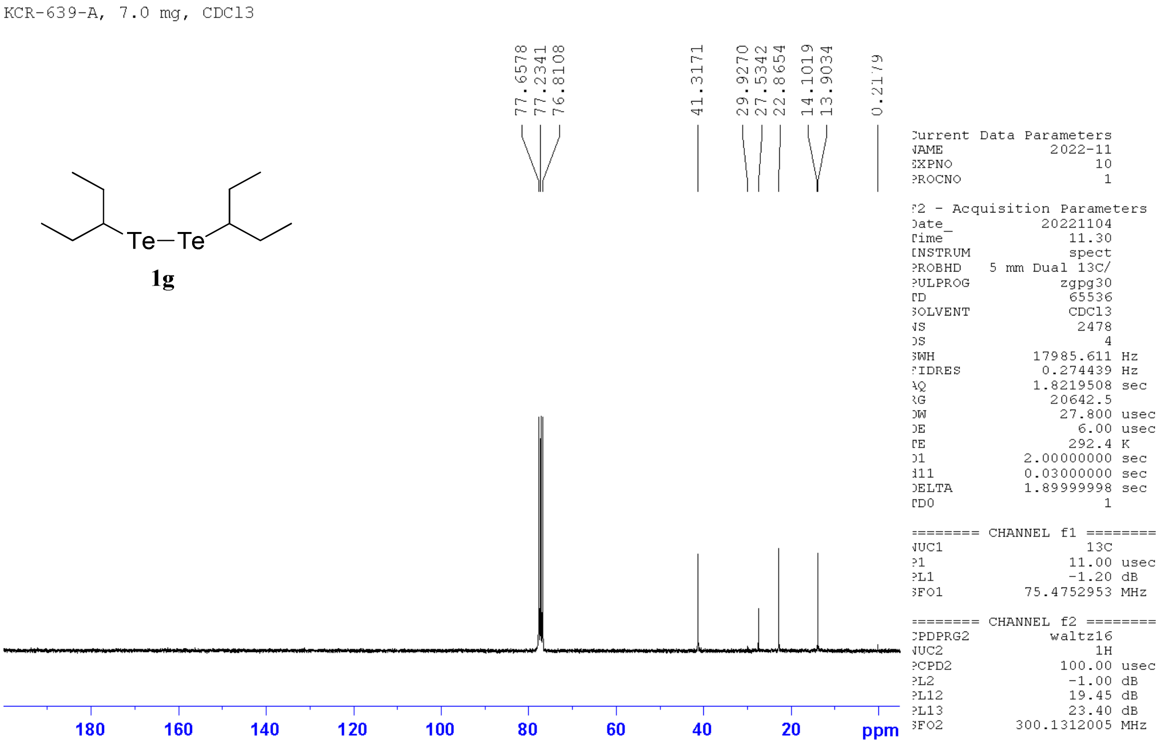

Figure S7b. ^{13}C NMR spectrum (75 MHz, $CDCl_3$) of compound **1g.**

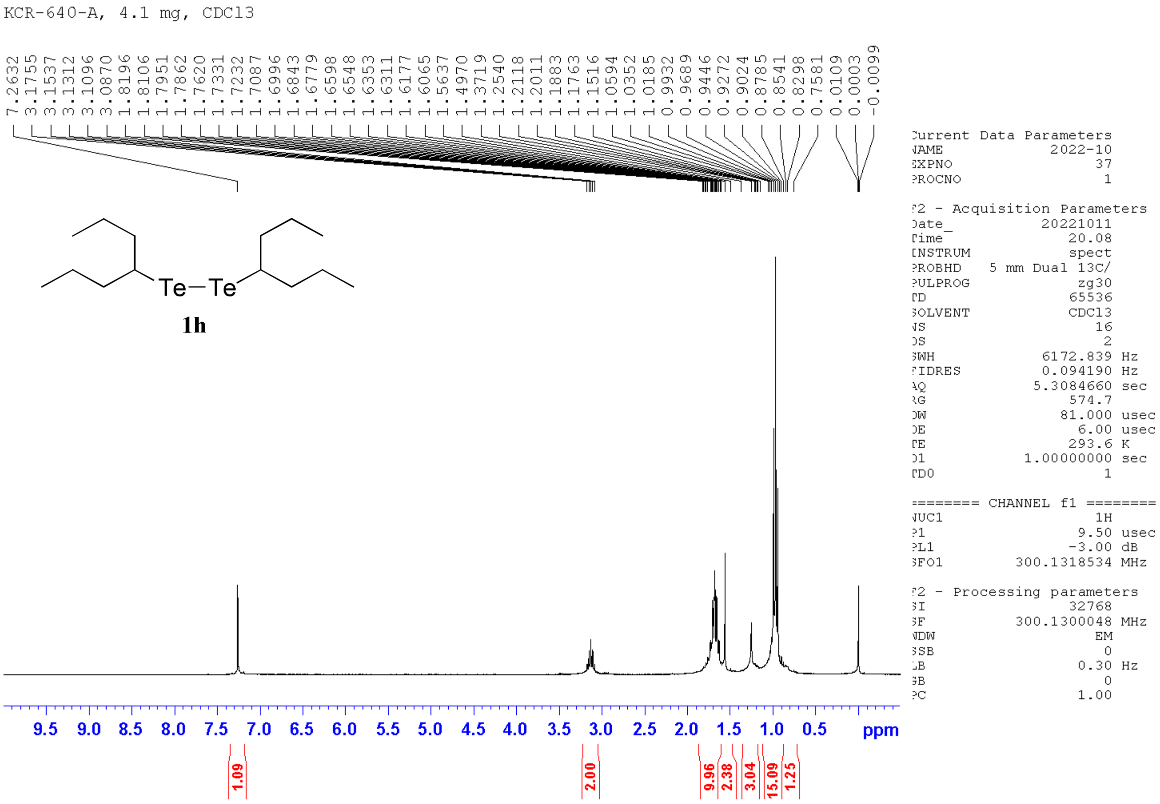

Figure S8a. ^{1}H NMR spectrum (300 MHz, $CDCl_3$) of compound **1h.**

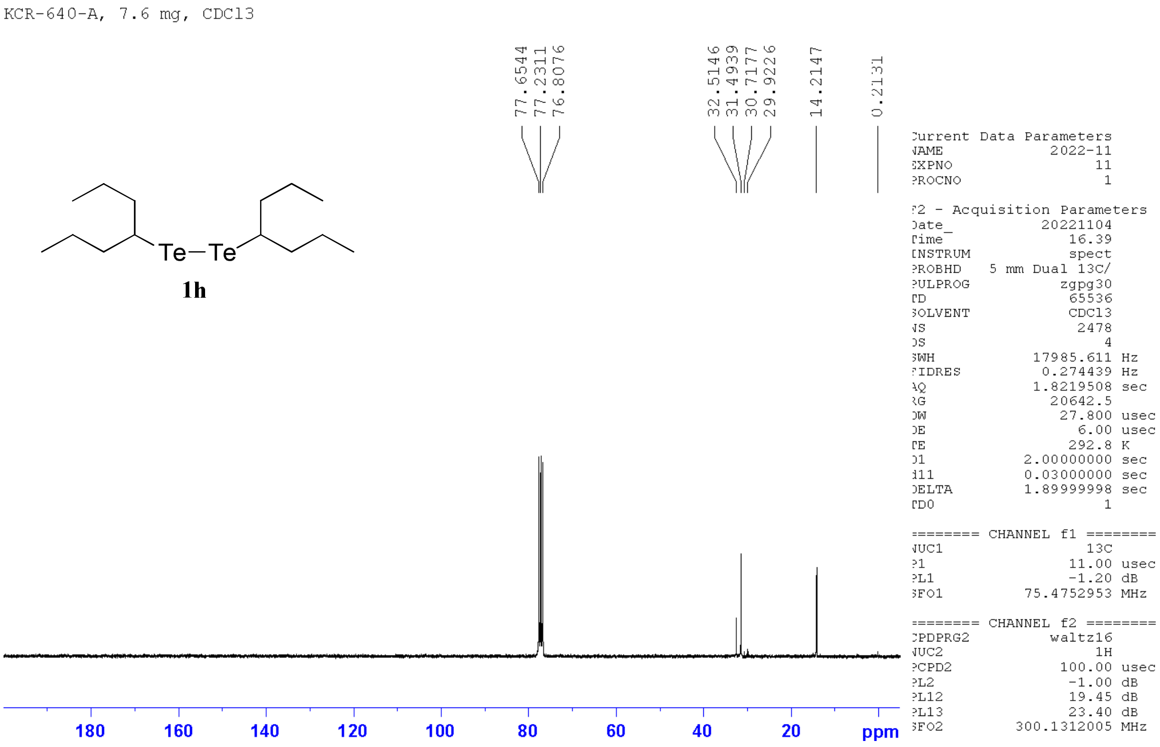

Figure S8b. ^{13}C NMR spectrum (75 MHz, $CDCl_3$) of compound **1h.**

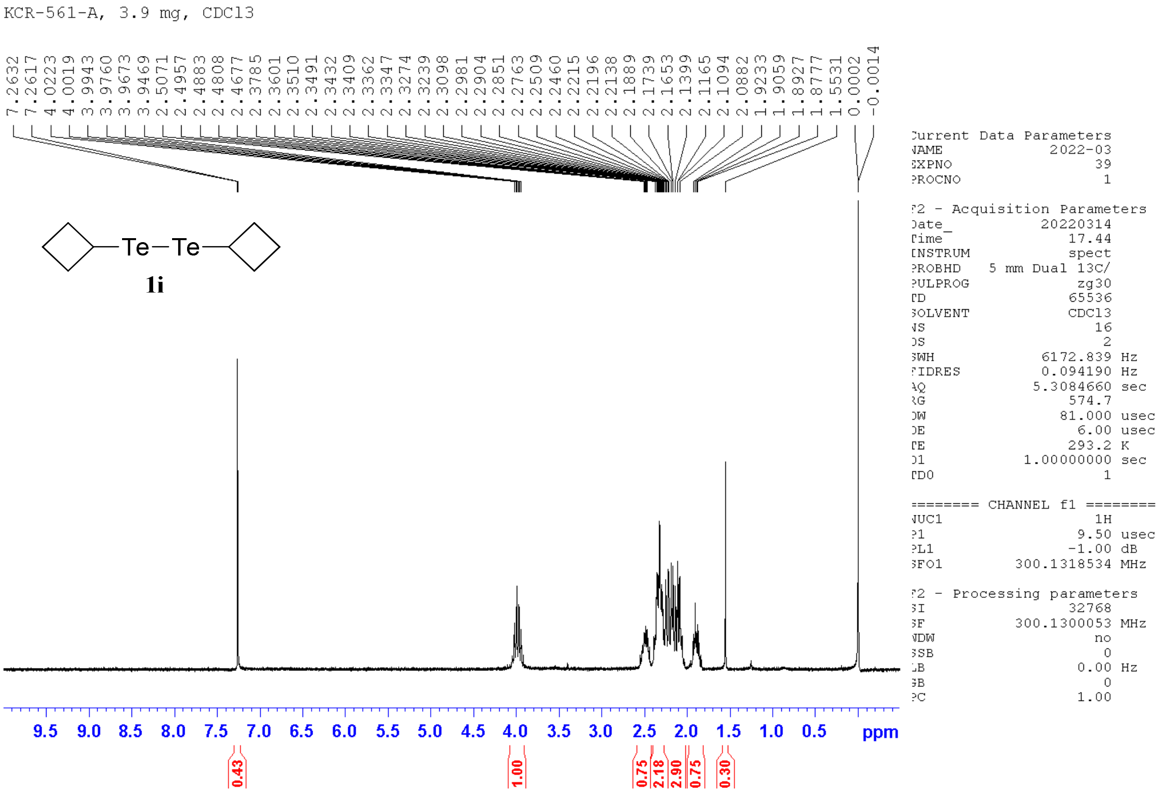

Figure S9a. ^{1}H NMR spectrum (300 MHz, $CDCl_3$) of compound **1i.**

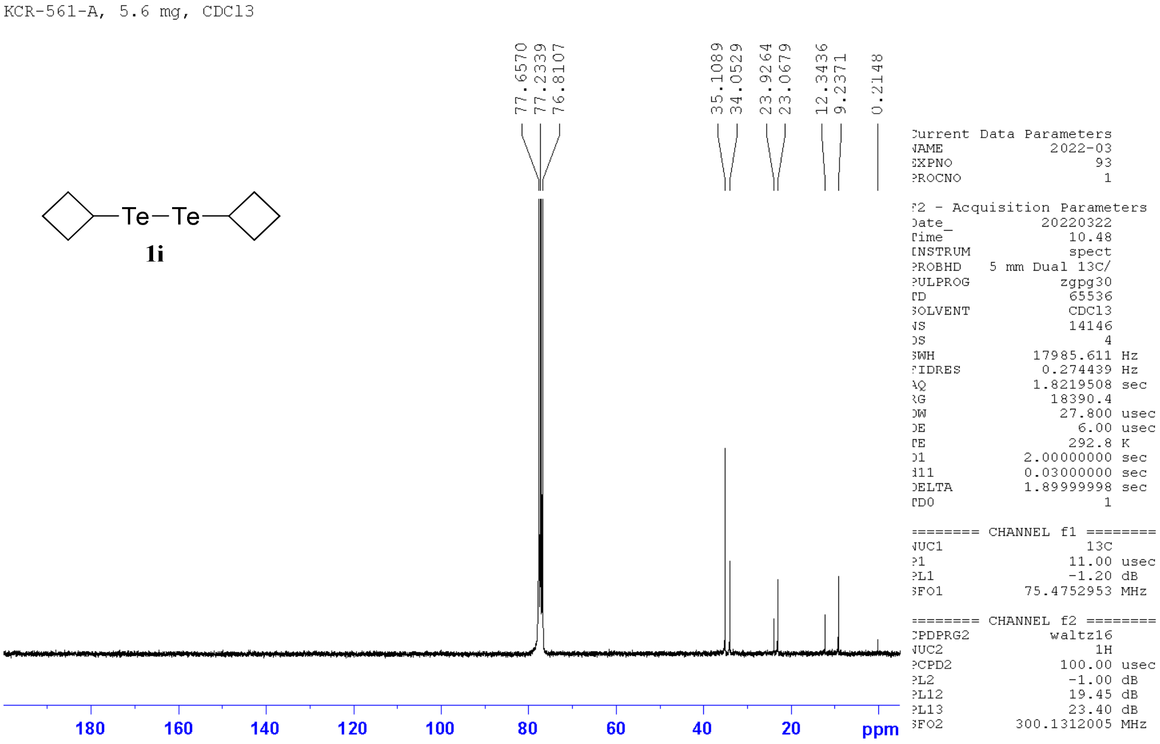

Figure S9b. ^{13}C NMR spectrum (75 MHz, $CDCl_3$) of compound **1i.**

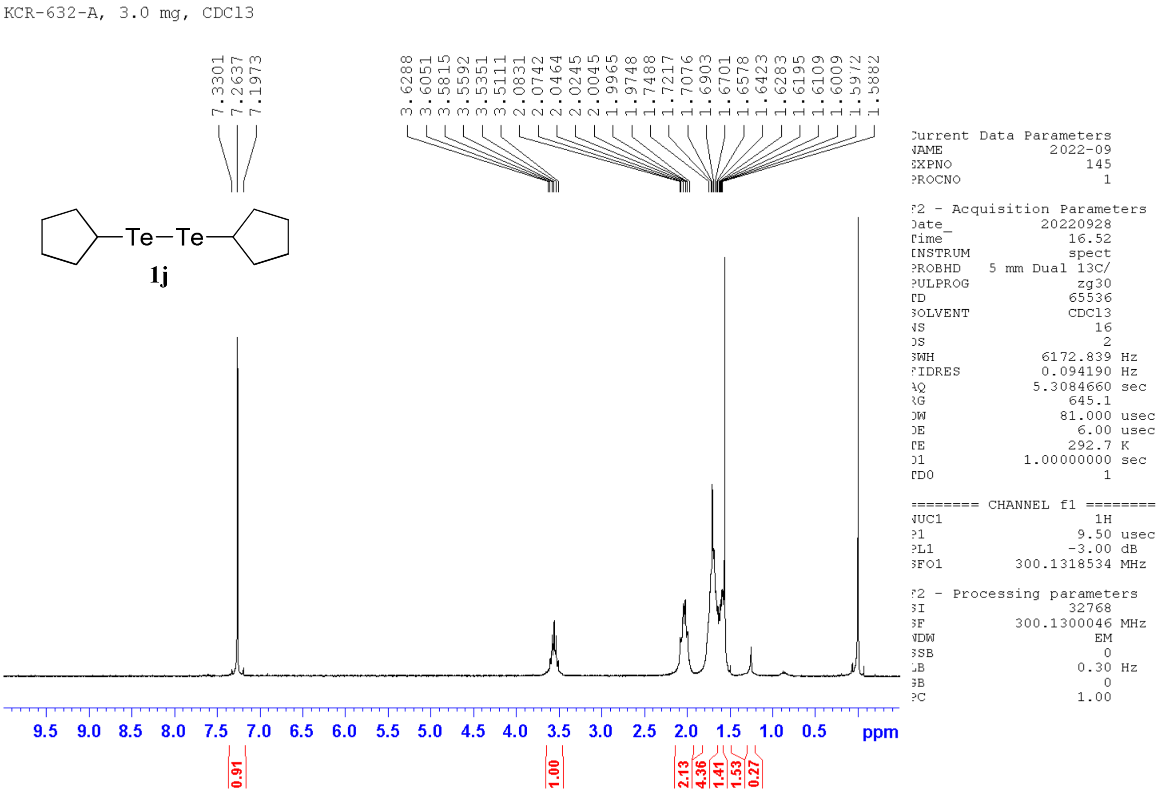

Figure S10a. ^{1}H NMR spectrum (300 MHz, $CDCl_3$) of compound **1j.**

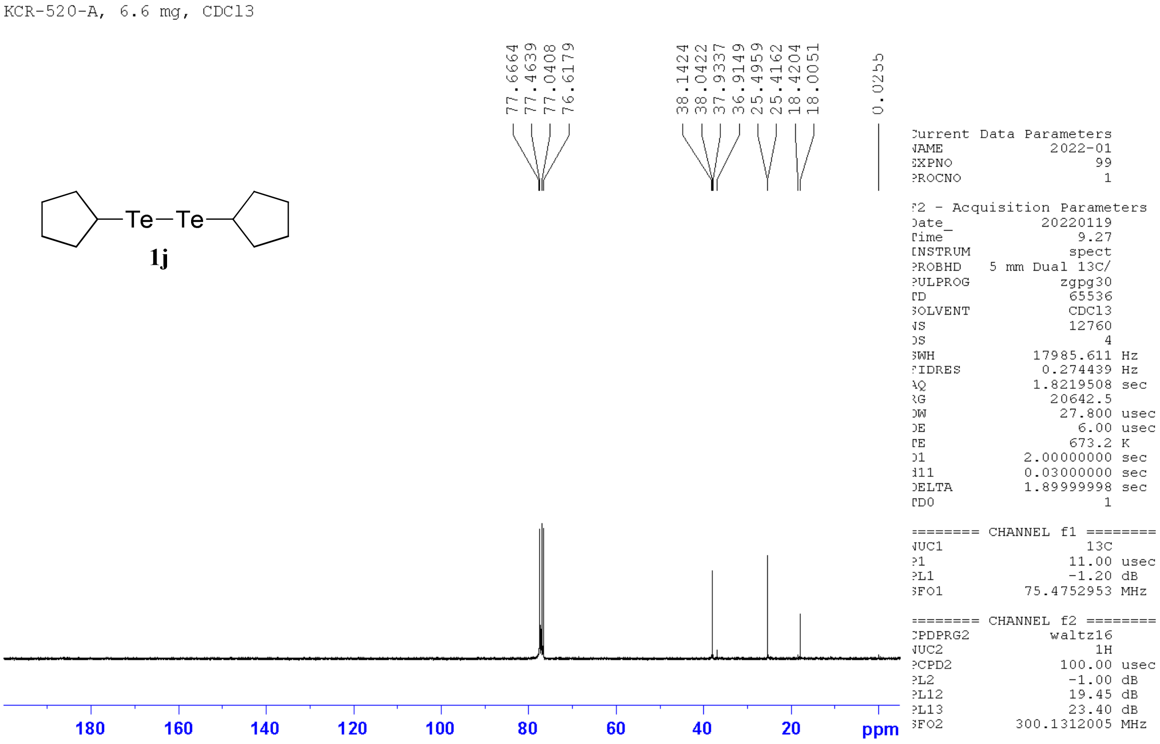

Figure S10b. ^{13}C NMR spectrum (75 MHz, $CDCl_3$) of compound **1j.**

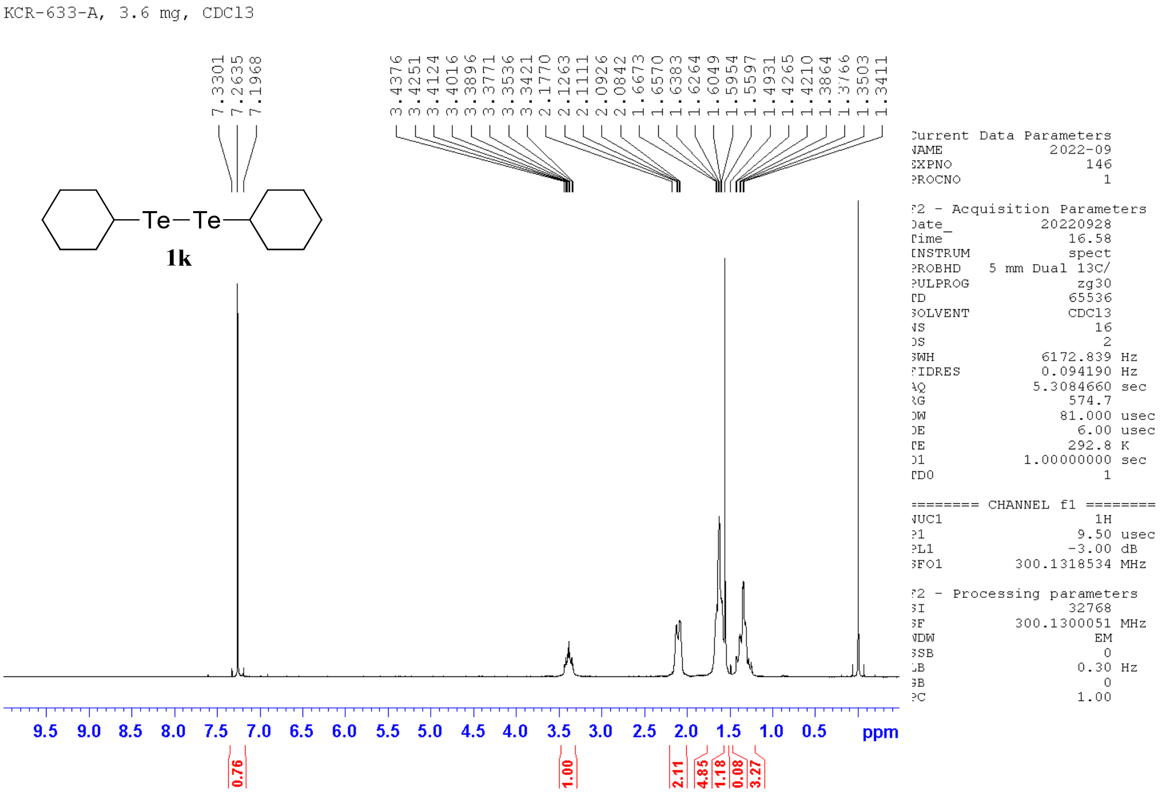

Figure S11a. ^{1}H NMR spectrum (300 MHz, $CDCl_3$) of compound **1k.**

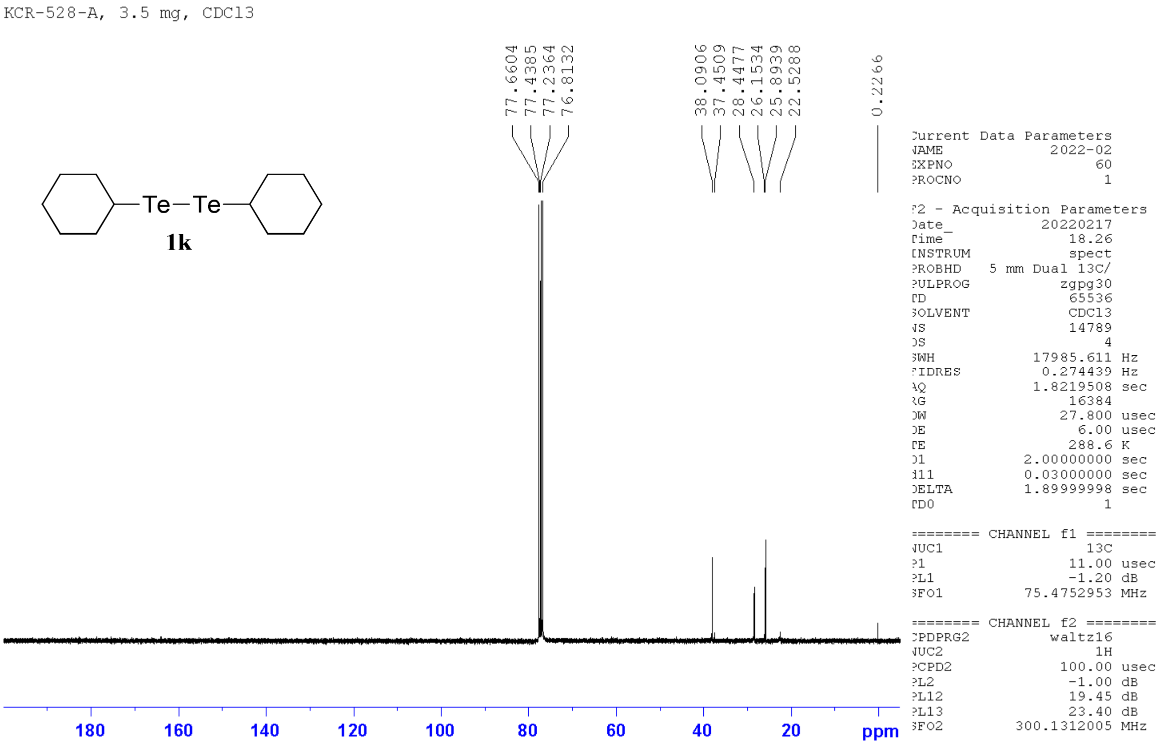

Figure S11b. ^{13}C NMR spectrum (75 MHz, $CDCl_3$) of compound **1k.**

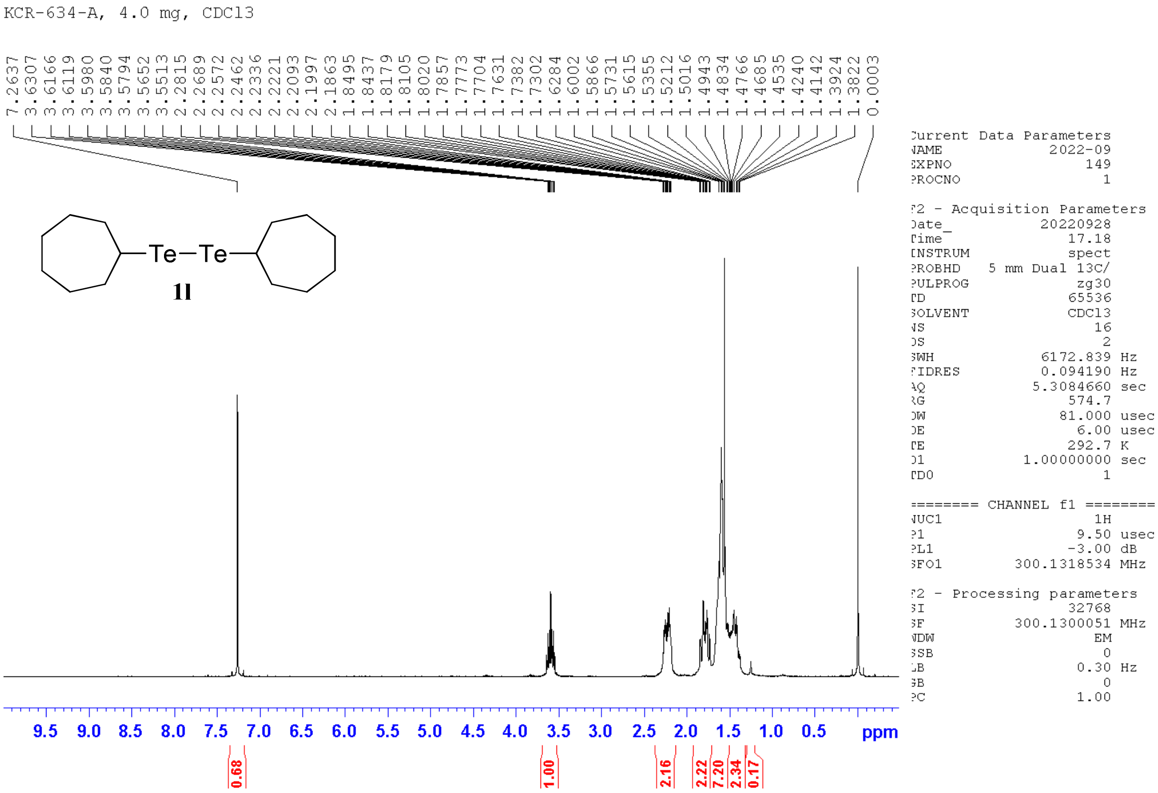

Figure S12a. ^{1}H NMR spectrum (300 MHz, $CDCl_3$) of compound **1l.**

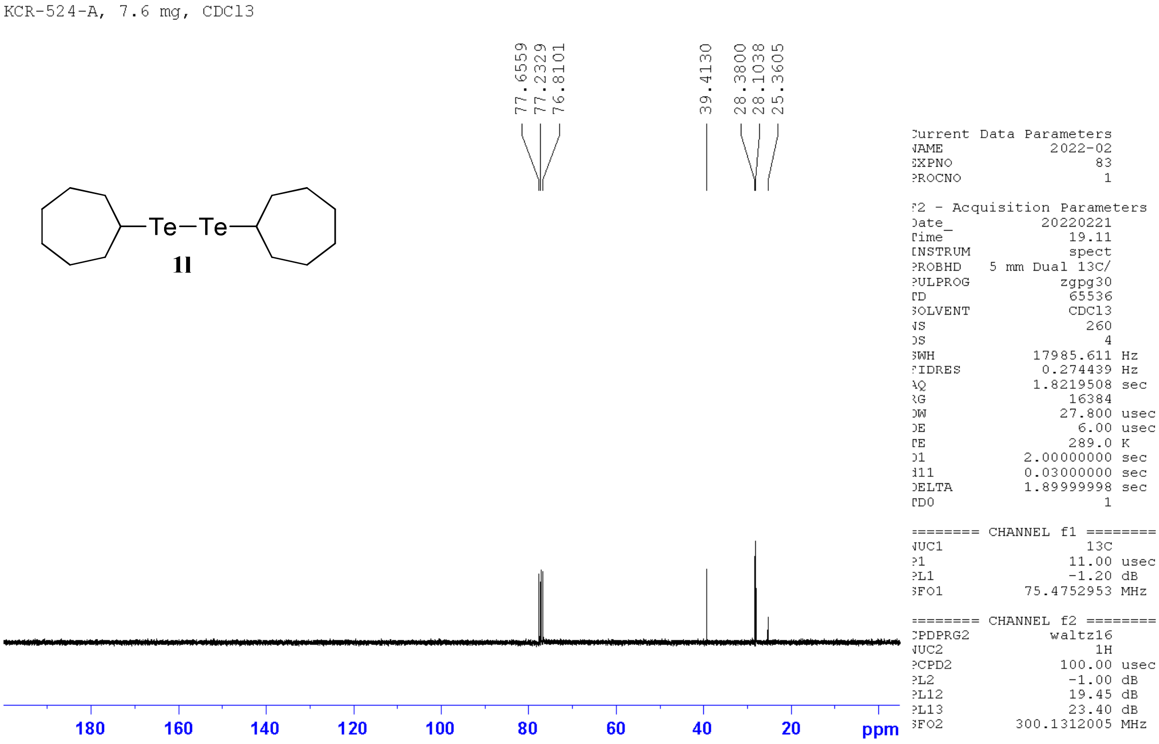

Figure S12b. ^{13}C NMR spectrum (75 MHz, $CDCl_3$) of compound **1l.**

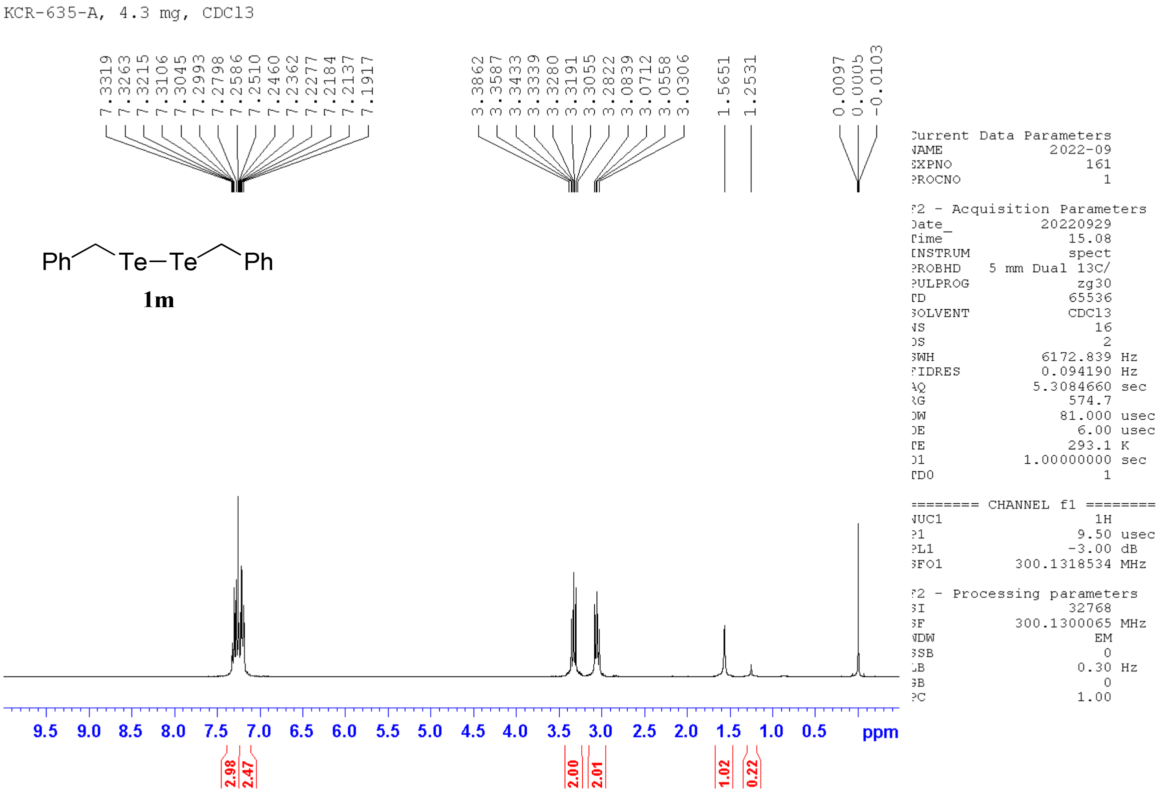

Figure S13a. ^{1}H NMR spectrum (300 MHz, $CDCl_3$) of compound **1m.**

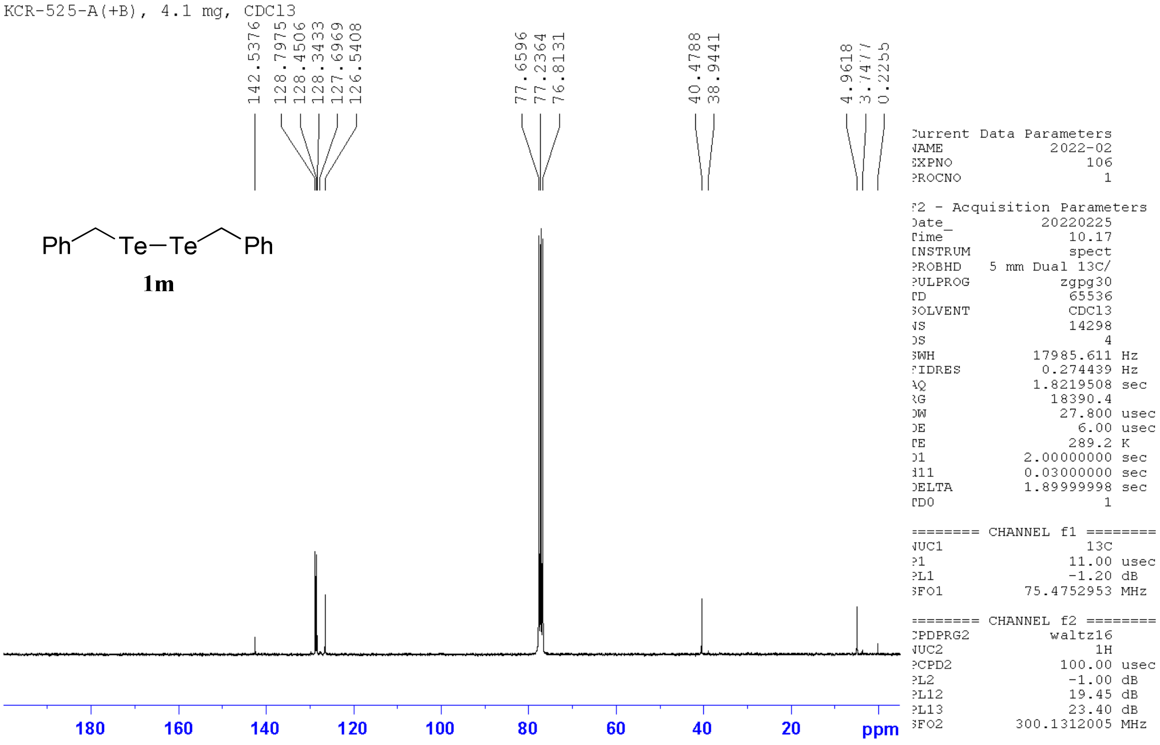

Figure S13b. ^{13}C NMR spectrum (75 MHz, $CDCl_3$) of compound **1m.**
